# Single-cell mutational burden distributions in birth–death processes

**DOI:** 10.1371/journal.pcbi.1013241

**Published:** 2025-07-07

**Authors:** Christo Morison, Dudley Stark, Weini Huang

**Affiliations:** 1 School of Mathematical Sciences, Queen Mary University of London, London, United Kingdom; 2 Group of Theoretical Biology, Research Section of Genomics, School of Life Sciences, Sun Yat-sen University, Guangzhou, China; University of Washington, UNITED STATES OF AMERICA

## Abstract

Genetic mutations are footprints of cancer evolution and reveal critical dynamic parameters of tumour growth, which otherwise are hard to measure *in vivo*. The mutation accumulation in tumour cell populations has been described by various statistics, such as site frequency spectra (SFS), single-cell division distributions (DD) and mutational burden distributions (MBD). While DD and SFS have been intensively studied in phylogenetics especially after the development of whole genome sequencing technology of bulk samples, MBD has drawn attention more recently with the single-cell sequencing data. Although those statistics all arise from the same somatic evolutionary process, an integrated understanding of these distributions is missing and requires novel mathematical tools to better inform the ecological and evolutionary dynamics of tumours. Here we introduce dynamical matrices to analyse and unite the SFS, DD and MBD and derive recurrence relations for the expectations of these three distributions. While we successfully recover classic exact results in pure-birth cases for the SFS and the DD through our new framework, we derive a new expression for the MBD and approximate all three distributions when death is introduced. We demonstrate a natural link between the SFS and the single-cell MBD, and show that the MBD can be regenerated through the DD. Counter-intuitively, the single-cell MBD is mainly driven by the stochasticity arising in the DD, rather than the extra stochasticity in the number of mutations at each cell division.

## Author summary

Somatic mutations accumulated in tissue growth and maintenance lead to genetic variation in tumours and healthy tissues. The patterns of those mutations have been used to reveal tumour history. Here, we developed a general framework to unite different statistical properties of mutation distributions between bulk sequencing data and single-cell data. The site frequency spectra from bulk data, division distributions and single-cell mutational burden distributions from single-cell data can be connected using dynamic matrices and recurrence relations. Counter-intuitively, the stochasticity in the number of mutations acquired in each cell division does not play a critical role in the single-cell mutation burden distribution.

## Introduction

Somatic mutations are important for the evolution of biological systems with clonal reproduction, including the development from healthy tissues to cancer [1,2]. While less is known about the somatic mutation rates in clonal species such as plants and corals, they have been studied extensively in human tissues. Healthy cells may accumulate in the order of 1 to 2 mutations per cell per division, which is directly observable in early development [3–5]. The mutational rate of tumour cells is often thought to be higher, which can be caused for example by genomic instability [6–8]. This large number of mutations accumulated in tumours serves as a genetic footprint to reveal their evolutionary history. Since the majority of these mutations are neutral [9], not impacting the fitness of a cell compared to its parental cell, neutral theory has been used to explain mutational patterns in many patient samples across different tumour types [10–12]. These measurements often demonstrate an early expansion of tumour populations, wherein driver mutations are clonal and the intratumour heterogeneity arises from neutral passenger mutations accumulated after cancer initiation. Although clonal interference, where cells carrying different sets of driver mutations intercompete, is a likely alternative scenario especially in large populations [13,14], here we focus on a further understanding of mutation accumulation under neutral selection as an important baseline dynamics.

Distributions of genetic heterogeneity under neutral selection have been studied in population genetics for over half a century [15,16]. One of such statistics is the site frequency spectrum (SFS), which describes the frequencies of mutations in a population [17]. Because the SFS deals with population-level information, it can be compared to bulk genomic data or pooled singe-cell data [12,18,19]. For an exponentially-growing population, a rescaling of the SFS, the variant allele frequency spectrum, has been shown to follow a $1/f^{2}$ power law, for *f* the frequency of a mutation in the population [12,18,20,21]. More recently, exact expressions for the SFS were found under the assumption of neutral evolution [22].

The advent of single-cell sequencing [23,24] opens the door for combining bulk and single-cell data to understand the growth history and dynamic traits of (healthy or tumorous) tissues, which are otherwise difficult to measure directly [19,25]. There is a great need for new mathematical and computational machinery to cope with single-cell data, which provides different mutational distributions beyond the SFS. The number of unique mutations in the population, also known as the overall tumour mutational burden (TMB) [26], has been studied both in a single tumour [22] and (its distribution) between tumours [27,28]. However, the distribution of mutational burdens between cells, the so-called single-cell mutational burden distribution (MBD), has only recently been experimentally observable through single-cell sequencing. Understanding the MBD may further help in inferring important evolutionary parameters, determining the growth history of the tumour and the level of selection at play, with neutral selection as a baseline with which to compare. Using data from healthy haematopoietic stem cells and oesophageal epithelial cells, Moeller, Mon Père *et al*. showed that analysis of single-cell and bulk data complement each other and narrowed down the parameter inference of the mutation rate and stem cell population size [19]. More specifically, the mean and variance of the MBD for a growing population were derived and used to estimate the underlying mutation rate [19]. However, the exact analytical shape of the MBD has not yet been explicitly found.

The MBD evolves during the cell division process, and thus an instructive object to study it with is the cell lineage tree [29], whose leaves symbolise living cells and whose root is the progenitor of the population. Branching processes can then be viewed as growing trees, where cell division is represented by a leaf bifurcating into two leaves, and cell death is the removal of a leaf. Because this framework can generate phylogenetic trees [30], cell lineage trees have properties that have been extensively studied [31]. One such property is the distribution of leaf heights (or the distances in edges from root to leaves), known as the division distribution (DD) of individual cells. By including the accumulation of new mutations at internal nodes of the tree, the MBD is obtained [29]. An expression for the DD generated by a pure-birth, or Yule, process has been found [32]; though when death is included it has not yet been solved exactly. Our goal is to build upon knowledge of the DD and the SFS to better understand the MBD, by formulating a discrete-time approach that integrates all three distributions.

We introduce a new framework via dynamical matrices to investigate mutation accumulation in a birth–death process and explain how key quantities such as the SFS, DD and MBD are obtained from these mutational matrices. This framework allows us to derive exact solutions of these distributions by recurrence relations in the pure-birth case, as well as first-order approximations when death is introduced, which hold in the low-death and large-population limits. By comparing our solutions for the SFS and DD to known results in population genetics, we first demonstrate the efficacy of our framework. We then show new results in expressions for the birth–death DD (Eq 7) and both the pure-birth and birth–death MBD (Eq 9). Our analytical results for all three distributions agree well with stochastic simulations. We find that the MBD can be generated via the DD and the mean mutation rate per cell division, independently of the stochasticity in the number of mutations per cell division.

## Results

We begin by describing a birth–death process with stochastic mutation accumulation, before deriving expected distributions for various summary statistics of interest.

### Dynamical matrices to unite the SFS, DD and MBD in a birth–death process

**Fig 1:**
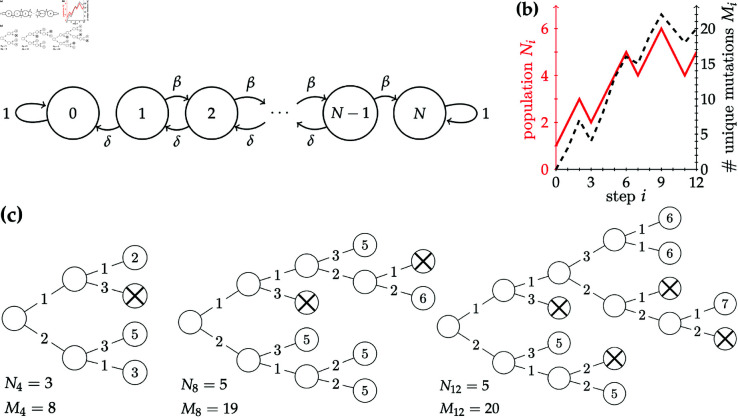
Discrete-time Markov chain model with binary tree representation of a sample realisation. **(a)** Discrete-time Markov chain description of the population size. **(b)** The population size *N*_*i*_ (solid red line) and number of unique mutations *M*_*i*_ (dashed black line) versus the step count *i* for the example realisation in (c). **(c)** Growing binary tree representations of an example realisation of the birth–death process with mutations described in the main text, with birth probability β=2/3, death probability δ=1/3, mutational mean μ=2, initial population *N*_0_ = 1. Cells that are crossed out have died. Edges are labelled by the number of new mutations occurring during that division. Leaves (living cells) are labelled by their mutational burden, which is equal to the sum of the edges that connect them to the root, or the mutation-free progenitor cell. The three sub-panels show snapshots of the process at steps *i* = 4, *i* = 8 and *i* = 12, with their population sizes *N*_*i*_ and *M*_*i*_ labelled.

In a birth–death process where a uniformly randomly chosen cell either divides with probability β or dies with probability δ=1−β, the population size *N*_*i*_ at time step *i* can be described by a discrete-time Markov chain (Fig 1a). The state space in this Markov chain is the finite integer set {0,…,N}, where *N* is the largest possible population size. In some cases, we are interested in the limit N→∞.

Often, the stochastic birth–death process explicitly involves a continuous time parameter *t* instead of a discrete step count *i*. This allows for rates to be considered instead of probabilities; however, as long as events are assumed not to be simultaneous, these two schemes can be mapped to one another by choosing a distribution of times between events. Most often, events are assumed to be exponentially distributed, and thus their frequencies grow with the population size.

Here, we focus on the growing-population case β>δ and assume *N*_0_ = 1 unless otherwise mentioned. In this case, the birth–death process is a growing rooted binary tree, where the root is the lone progenitor cell (assumed to be mutation-free, as any of its mutations will be clonal in the population), leaves are living cells, and pruned leaves are dead cells. See Fig 1b for an example realisation of such a process. Novel mutations are accumulated during cell divisions and old mutations may be lost in cell death. We deem mutations unique under the infinite sites approximation, where the probability of two point mutations occurring at the same location along the large genome is supposed vanishingly small [33]. Note that the infinite sites approximation has been disputed in the cancer context: Kuipers *et al*. [34] presented data that called into question the rarity of multiply-mutated sites; see Cheek and Antal [35,36] for branching process models that do not rely on the infinite sites approximation. Point mutations occurring during each duplication of the genome are modelled as occurring with a constant rate and independently from one another, a common modelling assumption [19]. Their occurrences therefore follow a Poisson process, and their number is Poisson distributed. Thus, when a cell divides, its daughter cells inherit all mutations carried by the mother cell and acquire a random number of new mutations drawn independently from a Poisson distribution with mean μ: $U_{1},U_{2}\sim \text{Pois}(\mu )$, where the indices refer to the two daughter cells.

We are most interested in three key quantities of this birth–death process with mutations at each step *i*: (i) the site frequency spectrum (SFS) $\{S_{j,i}{\}}_{j}$, whose elements *S*_*j*,*i*_ denote the number of mutations which occur *j* times in the population [17]; (ii) the single-cell mutational burden distribution (MBD) $\{B_{k,i}{\}}_{k}$, whose elements *B*_*k*,*i*_ are the number of cells having a mutational burden of *k* [19]; and (iii) the division distribution (DD) $\{D_{\ell ,i}{\}}_{\ell }$, whose elements $D_{\ell ,i}$ indicate the number of cells having undergone ℓ divisions during the process. Note that $D_{\ell ,i}$ is also the number of leaves lying at a distance of ℓ edges from the root in the growing tree framework.

While the importance of the SFS and the DD have been investigated in growing populations [12,18,20–22,31], we are interested in the relationship between them and how it can help us understand the MBD. Here, we introduce a novel discrete-time framework to demonstrate the symmetry between these distributions. The number of unique mutations at step *i* is $M_{i}=\sum_{j}S_{j,i}$, and the population size is $N_{i}=\sum_{k}B_{k,i}=\sum_{\ell }D_{\ell ,i}$, both of which are plotted in Fig 1c for the example found in Fig 1b. Thus, the MBD and the DD form partitions of the number of cells in a way similar to the SFS partitioning the number of unique mutations. Next, we introduce dynamical matrices to connect those quantities arising from the same population of individual cells.

**Fig 2:**
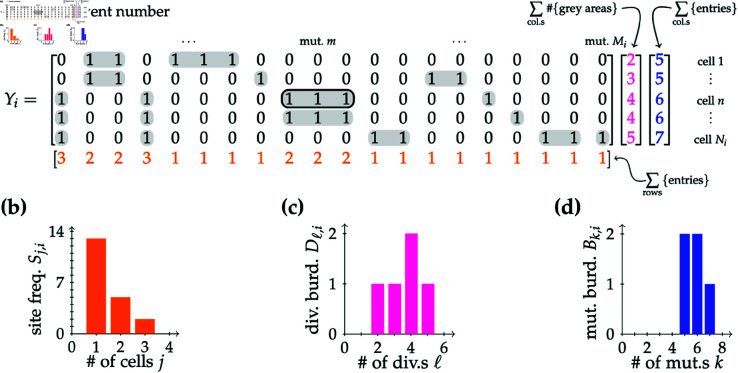
Dynamical matrix representation of the model with sample plots of the SFS, DD and MBD. The matrix framework described in the main text, where *i* refers to the Markov step count (in this example, *i* = 12). **(a)** The matrix *Y*_*i*_ corresponding to the example realisation of the birth–death process depicted in Fig 1b, where entry (*n*,*m*) is 1 if cell *n* possesses mutation *m* and 0 otherwise. Grey shaded mutational entries arose during the same division and thus always occur together in the descendants of their progenitor. For example, mutations *m*–1, *m* and *m* + 1 in cell *n* (shown outlined in black) were generated in the same past division. (In Fig 1b, we can determine by inspection this ancestor cell to be the one that later divided into two cells with mutational burdens of 6.) The row sum of the entries of *Y*_*i*_ is shown in orange, the column sum of the number of grey areas is in pink and the column sum of the entries of *Y*_*i*_ is in blue. **(b)** The site frequency spectrum (SFS) $\{S_{j,i}{\}}_{j}$: a histogram of the bottommost (orange) vector of (a). **(c)** The division distribution (DD) $\{D_{\ell ,i}{\}}_{\ell }$: a histogram of the middle (pink) vector of (a). **(d)** The single-cell mutational burden distribution (MBD) $\{B_{k,i}{\}}_{k}$: a histogram of the rightmost (blue) vector of (a), or equivalently of the sums of the weights of the edges in Fig 1b from the root to the leaves.

We consider a collection of matrices *Y*_*i*_, where the rows refer to cells and the columns refer to mutations, known as genotype matrices or SNP (single nucleotide polymorphism) matrices in bioinformatics [37]. Our matrices are dynamical in that their entries are updated at each step by a binary filling in the following manner: the (*n*,*m*)th entry of the matrix is equal to 1 if the *n*th cell possesses the *m*th mutation at step *i* and equal to 0 otherwise. When a cell dies, its row is removed from the matrix. Fig 2a shows an example of the matrix *Y*_*i*_ associated to the tree example in Fig 1b. We extend the concept of genotype matrices by marking mutations arising during a single (past) division by grey shaded areas. Note that if no mutations arise during a division, a placeholder column must be added with only zeros. The 0 entry corresponding to the cell that did not gain any new mutations would still be shaded in grey, as this shading tracks the division burden. These matrices are also how mutational data can be stored in stochastic simulations.

We can obtain the distributions of our key quantities, the SFS, DD and MBD (Fig 2b and 2d), from our dynamical mutation matrices (Fig 2a). For each mutation (column), the number of cells carrying this mutation is the row sum of the entries of *Y*_*i*_ (orange vector in Fig 2a). Thus, the histogram of this vector is the SFS at step *i*. For each cell (row), the number of divisions that the cell has undergone is the column sum of the number of grey areas (pink vector in Fig 2a), and the number of mutations in this cell is the column sum of the entries of *Y*_*i*_ (blue vector in Fig 2a). Correspondingly, the histograms of these two vectors lead to the other distributions obtainable from single-cell information: the DD and the MBD.

The symmetry provided by this mutation matrix *Y*_*i*_ gives rise to the following relationship between the site frequency spectrum and the single-cell mutational burden distribution:

$$\sum_{j=1}^{N_{i}}jS_{j,i}=\sum_{k=0}^{M_{i}}kB_{k,i}.$$ (1)

We call this quantity the number of mutational occurrences: that is, the sum of the entries of *Y*_*i*_.

### Law of total expectation and conditioning on survival

Our primary approach for deriving the distributions of our key quantities from the discrete-time model is as follows. We use the law of total expectation (𝔼[X]=𝔼[𝔼[X|Y]], for any random variables *X* and *Y*) to equate an expected quantity at step *i*  +  1 to a conditional expectation. This usually is a function of the quantity at step *i* (conditional on knowledge of this information at step *i*), as earlier knowledge is never needed due to the Markov nature of the model. From this, we derive a recurrence relation for the expected values of our desired quantity, which can be solved.

We first note that conditioning on the survival of the entire population plays a role in all of our subsequent expected values. In the pure-birth case, the population is deterministic and equal to *N*_*i*_ = *i* + 1. Once death is included, however, the population size becomes a random variable. For the birth–death chain of Fig 1a, its expected value at step *i*, both conditioned on survival and not, can be exactly calculated, which is done in Proposition B of S1 Appendix. Fig A of S1 Appendix shows that the expected population size conditioned on survival can be linearly approximated by $𝔼\left[N_{i}\right]≃(\beta -\delta )i+1$, valid for low death, since in this limit the expected gain in population in one step is β − δ. All of our ensuing expectations are conditioned on survival and the initial condition *N*_0_ = 1, which we will omit from our notation for brevity. Table 1 displays our notation.

**Table 1:** Notation used in this manuscript.

Symbol	Description
β	Birth probability (Markov transition rate)
δ	Death probability (Markov transition rate)
*N*_*i*_	Population size at step *i*
*N*	Maximal population size
μ	Mutational mean: mean number of mutations acquired per division per daughter cell
*M*_*i*_	Number of unique mutations (tumour mutational burden, TMB) at step *i*
$\{S_{j,i}{\}}_{j}$	Site frequency spectrum (SFS), where *S*_*j*,*i*_ is the number of mutations occurring *j* times in the population at step *i*
$\{B_{k,i}{\}}_{k}$	Single-cell mutational burden distribution (MBD), where *B*_*k*,*i*_ is the number of cells with *k* mutations at step *i*
$\{D_{\ell ,i}{\}}_{\ell }$	Division distribution (DD), where $D_{\ell ,i}$ is the number of cells having undergone ℓ divisions at step *i*
*Y*_*i*_	Mutational matrix at step *i*: entry (*n*,*m*) is 1 if cell *n* possesses mutation *m*, 0 otherwise
[iℓ]	Unsigned Stirling number of the first kind with indices 1≤ℓ≤i

### Site frequency spectrum

With the recurrence relation method outlined above, we can formally derive the pure-birth (β=1) site frequency spectrum. For any birth probability β, the total instances of *j*-abundant mutations in the population at step *i* is equal to *jS*_*j*,*i*_. For example, in the leftmost sub-panel of Fig 1c, one can verify that there are 1 · *S*_1,4_ = 7 instances of 1-abundant mutations and 2 · *S*_2,4_ = 4 instances of 2-abundant mutations in the population at step *i* = 4. When there is no death, *N*_*i*_ = *i*  +  1. Thus, at step *i*, the expected number of *j*-abundant mutations in a (randomly chosen) dividing cell is *jS*_*j*,*i*_/(*i*  +  1), since we average the total number of instances over the population. After the division in step *i*, any *j*-abundant mutations in the dividing cell will become (j+1)-abundant and thus no longer contribute to the *j*-site. Similarly, (*j*–1)-abundant mutations in the dividing cell now contribute to the *j*-site. We therefore have


$$𝔼\left[S_{j,i+1}\right]=𝔼\left[𝔼\left[S_{j,i+1}\;|\;\{S_{j^{\prime },i}{\}}_{j^{\prime }}\right]\right]$$

$$=𝔼\left[S_{j,i}-\frac{jS_{j,i}}{i+1}+\frac{(j-1)S_{j-1,i}}{i+1}+\left(U_{1}+U_{2}\right)\delta_{1,j}\right],$$ (2)

for $\delta_{\cdot ,\cdot }$ the Kronecker delta symbol, whose term arises from the new 1-abundant mutations occurring during division, which are independently drawn from a Poisson distribution: $U_{1},U_{2}\sim \text{Pois}(\mu )$.

We make the change of variables $Q_{j}=𝔼\left[S_{j,i}\right]/(i$  +  1), absorbing the source term into the boundary condition $Q_{1}=\mu$. That *Q*_*j*_ is independent of *i* can be argued in the following manner: in expectation, during neutral exponential growth, mutations preserve their frequency in the population, as all cells (both those with and without a given mutation) grow at the same rate [12]. (For a brute-force demonstration of this, see the Supplementary Information of [19].) Thus, using the linearity of expectation, Eq (2) becomes simply $(j+1)Q_{j}=(j-1)Q_{j-1}$, which telescopes to obtain the known result


$$𝔼\left[S_{j,i}\right]=\frac{2\mu (i+1)}{j(j+1)}.$$

An identical procedure can be applied in the birth–death case to recover the large-population expected SFS, though a difficulty here is that now *N*_*i*_ becomes a random variable itself. This means that the denominators *i*  +  1 in Eq (2) become *N*_*i*_, and so we are left with terms of the form $𝔼\left[S_{•,•}/N_{•}\right]$.

When random variables *A* and nonzero *B* are close to their expected values, expanding 𝔼[A/B] around the point (𝔼[A],𝔼[B]) provides the first-order approximation 𝔼[A/B]≃𝔼[A]/𝔼[B] (see Corollary C of S1 Appendix). When we state *to first order*, this is the approximation we are making and the region of interest—near (𝔼[A],𝔼[B])—unless otherwise specified.

Here, we outline the birth–death derivation of the SFS, leaving the details for Proposition D of S1 Appendix. Let *R* (for “reaction”) be a random variable equal to 1 when a division event takes place (so that ℙ[R=1]=β) and –1 when a death event occurs (ℙ[R=−1]=δ). By conditioning on these two outcomes and multiplying their probabilities of occurrence, the law of total expectation provides the corresponding recurrence relation to Eq (2):


$$𝔼\left[S_{j,i+1}\right]=𝔼\left[𝔼\left[S_{j,i+1}\;|\;\{S_{j^{\prime },i}{\}}_{j^{\prime }\leq i}\right]\right]\;=𝔼[S_{j,i}+{𝟙}_{\left\{R=1\right\}}\left(-\frac{jS_{j,i}}{N_{i}}+\frac{(j-1)S_{j-1,i}}{N_{i}}+2\mu \delta_{1,j}\right)\;\;\;\;+{𝟙}_{\left\{R=-1\right\}}\left(-\frac{jS_{j,i}}{N_{i}}+\frac{(j+1)S_{j+1,i}}{N_{i}}\right)],$$

where we have used the indicator function ${𝟙}_{A}$ to be 1 on the set *A* and 0 elsewhere. Since *R* is independent from the other random variables at play, the expectation of the indication functions ${𝟙}_{\left\{R=\pm 1\right\}}$ become β and δ, respectively.

By expanding *to first order*, we can make the ansatz $𝔼\left[S_{j,i}/N_{i}\right]≃𝔼\left[S_{j,i}\right]/𝔼\left[N_{i}\right]=X_{j}$, as in the pure-birth case. After some rearranging, this produces a homogeneous second-order recurrence relation of the form $f_{2}X_{j+2}+f_{1}X_{j+1}+f_{0}X_{j}=0$, for some linear functions $\{f_{n}{\}}_{n=0}^{2}$ of *j*. Solutions of such recurrence relations are known [38], and we obtain the following first-order approximation, which matches the N→∞ result from [22]:

$$𝔼\left[S_{j,i}\right]≃\sum_{j^{\prime }=0}^{\infty }\frac{2\mu (\delta /\beta {)}^{j^{\prime }}}{\left(j+j^{\prime })(j+j^{\prime }+1\right)}𝔼\left[N_{i}\right],$$ (3)

where all expectations are conditioned on non-extinction of the whole population [22]. In the limit of low death (δ≪β), this approximation is sound, as then the variance in population size is small (see Proposition F of S1 Appendix for details).

### Division distribution

**Fig 3:**
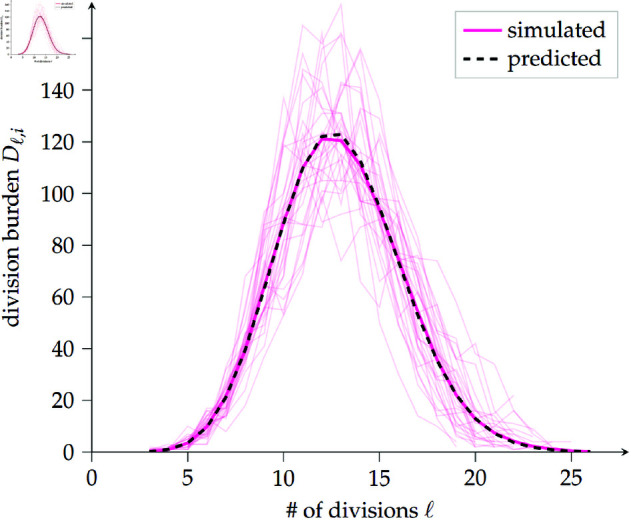
Expected division distribution for a pure-birth process matches stochastic simulation results. Average (solid dark pink line) of 200 simulation realisations (representatives in solid pale pink lines) of the division distribution (DD) for a pure-birth process up to final population size *N* = 10^3^, along with the predicted expected DD obtained from Eq (5) (dashed black line).

The expected division distribution in the pure-birth case can be obtained in a similar manner as the site frequency spectrum. The probability of selecting a cell with ℓ divisions in its history is $D_{\ell ,i}/(i\;+\;1)$: that is, the number of cells have divided ℓ times, divided by the total population. This factor will then no longer contribute to $D_{\ell ,i+1}$, dividing into two cells with one more division in their history than before. The law of total expectation then becomes

$$𝔼\left[D_{\ell ,i+1}\right]=𝔼\left[𝔼\left[D_{\ell ,i+1}\;|\;\{D_{\ell^{\prime },i}{\}}_{\ell^{\prime }\leq i}\right]\right]=𝔼\left[D_{\ell ,i}-\frac{D_{\ell ,i}}{i+1}+\frac{2D_{\ell -1,i}}{i+1}\right],$$ (4)

which can be solved to recover the result from [32]:

$$𝔼\left[D_{\ell ,i}\right]=\left[\begin{array}{c} i\\ \ell \end{array}\right]\frac{2^{\ell }}{i!},$$ (5)

where the unsigned Stirling numbers of the first kind [iℓ] are defined by the relation


$$\left[\begin{array}{c} i+1\\ \ell \end{array}\right]=i\left[\begin{array}{c} i\\ \ell \end{array}\right]+\left[\begin{array}{c} i\\ \ell -1 \end{array}\right]\;\text{ for }1\leq \ell \leq i,$$

with boundary conditions [11]=1 and [iℓ]=0 if ℓ>i or ℓ=0. Eq (5) can be substituted into Eq (4) to show that it satisfies the desired recurrence relation; by uniqueness of solutions that agree with boundary conditions, we have the result. Distributions generated by stochastic simulations using a Gillespie algorithm (see Methods) agree well with this expression (see Fig 3).

We outline the birth–death derivation of the DD, leaving the details to Proposition E of S1 Appendix. Again, for *R* the random variable denoting division or death, the δ>0 recurrence relation corresponding to Eq (4) can be written

$$𝔼\left[D_{\ell ,i+1}\right]=𝔼\left[D_{\ell ,i}+{𝟙}_{\left\{R=1\right\}}\left(-\frac{1}{N_{i}}D_{\ell ,i}+\frac{2}{N_{i}}D_{\ell -1,i}\right)+{𝟙}_{\left\{R=-1\right\}}\left(-\frac{1}{N_{i}}D_{\ell ,i}\right)\right].$$ (6)

Similarly to with the SFS, we can expand to first order: $𝔼\left[D_{\ell ,i}/N_{i}\right]≃𝔼\left[D_{\ell ,i}\right]/𝔼\left[N_{i}\right]$; this is valid near $\left(𝔼\left[D_{\ell ,i}\right],𝔼\left[N_{i}\right]\right)$, so the expansion will hold when $D_{\ell ,i}$ and *N*_*i*_ are close to their expected values. We also make the linear approximation $𝔼\left[N_{i}\right]≃(\beta -\delta )i+1$, valid for low death; see Fig A of S1 Appendix and surrounding remarks for details. This allows us to rewrite an ansatz 𝔼[Dℓ,i]az=[iℓ](2β)ℓ(β−δ)i−ℓ/∏i′=1i−1𝔼[Ni′], which can be shown to satisfy Eq (6), into the following neat first-order approximation for the birth–death DD:

𝔼[Dℓ,i]≃[iℓ]2ℓ(1−δ/β)−ℓ∑ℓ′=1i[iℓ′]2ℓ′(1−δ/β)−ℓ′𝔼[Ni]. (7)

Here, it is evident that the division distribution partitions the population size (since summing the fraction on the right-hand side over ℓ gives unity) and that this partitioning is orchestrated by the functions f(i,ℓ)=[iℓ]2ℓ(1−δ/β)−ℓ.

### Mutational burden distribution

**Fig 4:**
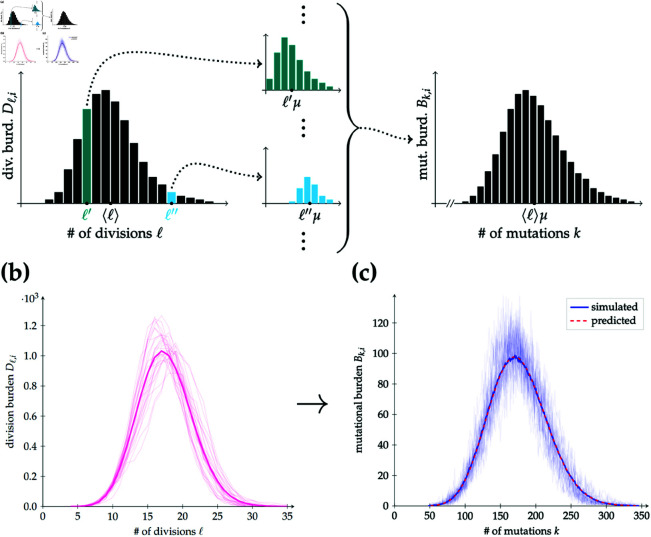
Conversion of the division distribution into the single-cell mutational burden distribution. **(a)** The elements of a DD are translated into Poisson distributions with means ℓμ, weighted by $D_{\ell ,i}$ (such that the sums over the teal and cyan distributions are $D_{\ell^{\prime },i}$ and $D_{\ell^{\prime \prime },i}$, respectively, for example), and then summed to obtain the corresponding MBD. Note that if the mean of the DD is ⟨ℓ⟩, then the mean of the resulting MBD is ⟨ℓ⟩μ. **(b)** Average (solid dark pink line) of 200 simulation realisations (representatives in solid pale pink lines) of the DD for a pure-birth process up to final population size *N* = 10^4^ with mutational mean μ=10. **(c)** Average (solid dark blue line) of the MBD for the same simulation realisations as (b) (representatives in solid pale blue lines), along with the MBD obtained from converting the average DD as explained in (a) and the main text (dashed red line).

The single-cell mutational burden distribution differs from the division distribution because there is an additional stochasticity at each cell division due to mutational (Poisson) distributions. To obtain an expected MBD from a DD, we can employ a procedure to introduce this stochasticity as follows: each cell contributing to a division burden $D_{\ell ,i}$ will have undergone ℓ divisions, so will have acquired $\sum_{p=1}^{\ell }U_{p}$ mutations, where $U_{p}\sim \text{Pois}(\mu )$ represents the number of mutations acquired during the cell’s *p*th division. Since the Poisson distribution is additive, this sum is in turn a Poisson-distributed random variable with mean ℓμ. The left-hand side of Fig 4a qualitatively depicts the elements of the DD being converted into Poisson probability mass functions associated to these sums of Poisson variables. These are then summed to obtain the MBD, shown on the right-hand side of Fig 4a, in the following manner.

Writing $U_{p,q,\ell }\sim \text{Pois}(\mu )$ for the number of mutations acquired during the *p*th division of the *q*th cell (for some labelling of cells $1\leq q\leq D_{\ell ,i}$) having undergone ℓ divisions, we can sum over the elements of the DD labelled by ℓ to obtain

$$𝔼\left[B_{k,i}\right]=𝔼\left[𝔼\left[B_{k,i}\;|\;\{D_{\ell^{\prime },i}{\}}_{\ell^{\prime }\leq i}\right]\right]=𝔼\left[𝔼\left[\sum_{\ell =0}^{i}\sum_{q=1}^{D_{\ell ,i}}{𝟙}_{\left\{\sum_{p=1}^{\ell }U_{p,q,\ell }=k\right\}}\;|\;\{D_{\ell^{\prime },i}{\}}_{\ell^{\prime }}\right]\right].$$ (8)

Now, using the linearity of expectation, that *i* is fixed and ℓ just an index, and the independence of the random variables $D_{\ell ,i}$ and $U_{p,q,\ell }$, the right-hand side of Eq (8) becomes


$$\sum_{\ell =0}^{i}𝔼\left[D_{\ell ,i}𝔼\left[{𝟙}_{\left\{\sum_{p=1}^{\ell }U_{p,q,\ell }=k\right\}}\right]\right]=\sum_{\ell =0}^{i}𝔼\left[D_{\ell ,i}\right]𝔼\left[{𝟙}_{\left\{\sum_{p=1}^{\ell }U_{p,q,\ell }=k\right\}}\right].$$

Finally, substituting the expression in Eq 5 for the expected DD and the probability mass function for the Poisson distribution with mean ℓμ, we find the pure-birth expected MBD:

$$𝔼\left[B_{k,i}\right]=\sum_{\ell =0}^{i}\left[\begin{array}{c} i\\ \ell \end{array}\right]\frac{2^{\ell }}{i!}\frac{e^{-\ell \mu }(\ell \mu {)}^{k}}{k!}.$$ (9)

Fig 4b and 4c verify the conversion from a DD to a MBD described in the previous discussion with simulations.

The same conversion procedure can be implemented in the birth–death case. Again, working with first-order approximations, the expression in Eq (7) for the expected birth–death DD can be used instead of the pure-birth expression in Eq (5) during the final step to obtain a first-order approximation of the expected birth–death MBD.

Finally, consider the number of mutational occurrences: that is, the sum of the entries of the mutational matrix *Y*_*i*_ or, equivalently, either side of Eq (1). If this quantity is divided by the number of mutations *M*_*i*_, we obtain the mean of the SFS; and if it is divided by the population *N*_*i*_, we obtain the mean of the MBD. We can derive the expected number of mutational occurrences using our recurrence relation approach, from which we deduce that this mean, representing the expected mutational burden of a cell, grows logarithmically with the step count *i* (see Propositions G and H of S1 Appendix). In the pure-birth case, it is simply a rescaling of the harmonic numbers.

## Discussion

The distribution of genetic mutations in cell populations has been studied both in the cases of constant [17,19,37,39] and growing populations [12,21,40–45]. With the development of single-cell sequencing technologies, exploration of more precise information in single cells is sure to follow in the footsteps of population-level research [23,24,46]. At the population level, both site frequency spectra (SFS) and overall tumour mutational burden (TMB) have been investigated analytically [12,18–22]. Here we focus on the single-cell distribution of the latter (the single-cell mutational burden distribution, or MBD), and use the foundation of the SFS to better understand the MBD analytically.

A new framework uniting the SFS and the MBD is presented, relying on a simple procedure: dynamical matrices store the mutational information of a population of cells, whose size is dictated by a birth–death process. Our approach of encoding the data in binary matrices, where the entry (*n*,*m*) is 1 when cell *n* has mutation *m* and 0 otherwise, naturally emerges from the (neutral) evolution-motivated idea wherein a cell is identified by its mutation load [37]. Two different ways of partitioning the entries of this mutational matrix provide definitions of both the SFS and the MBD as histograms of the row- and column-sums, respectively, as shown in Fig 2. With this symmetry in mind, which gives rise to Eq (1), an identical analytical approach depending on the discrete-time Markov nature of the model can be applied to both cases, along with an intermediary case of the division distribution (DD), to obtain recurrence relations for the distributions of interest: we employ the law of total expectation to write the expected value of a quantity of interest in terms of expected values at the previous time step. These recurrences are solved exactly in the pure-birth case and approximately in the birth–death case, giving rise to analytical predictions for the SFS, DD and MBD, which are compared to stochastic simulations as well as previous work on the SFS and the DD.

Indeed, in Propositions D and H of S1 Appendix, we recover the expected values of the SFS and TMB derived by Gunnarsson *et al*. [22] (their Propositions 2 and 3). Our stochastic-time first-order approximation in Eq (3) matches theirs from the stochastic-population scenario with a fixed elapsed time in the large-population limit, where the regimes coincide according to their convergence analysis [22]. Our derivation for the pure-birth DD in Eq (5) recovers a result from previous work on phylogenetic trees produced by Yule processes: combinatorics results relating to binary search trees [32] were then applied to the phylogenetic context [31].

The reverse-time coalescent approach supplies complementary tools to branching processes (though more often compared to the continuous-time setting [47]), which can also provide information on the summary statistics we discuss here [48]. Coalescent theory allows one to reconstruct phylogenic trees that describe genetic information found in individuals sample at present. Phylogenetic tree branch lengths can then be used to determine the mutations accumulated during this time interval, along with informing the population growth rate [47]. For example, Popovic [49] introduced the coalescent point process (CPP), which reconstructs phylogenetic trees using independent and identically-distributed coalescent times for a sample of individuals; when populations’ genealogies can be represented with this formalism, their population size satisfies a geometric distribution [49]. Lambert [50] used the CPP to derive the expected SFS under certain conditions and later integrated the effects of sampling: both Lambert and Stadler [51] and Lambert [52] derived distributions of node depths of a sample within a phylogenetic tree. This relates to our DD—although, again, in a differently-conditioned, continuous-time process. Still more recently, Schweinsberg and Shuai [47] recovered the supercritical SFS result of Durrett [20] (that is, the 1/(j(j+1)) form in Eq (3)) using CPPs; with Johnson and Curtius, they applied these results to haematopoeitic data to infer growth rates of clones with one or multiple driver mutations [53]. While phylogenetic trees inferred using coalescent theory can be mapped to DDs—or, directly to the MBD, if every division event corresponds to a point of coalescence—to our knowledge the triple connection between the SFS, the DD and the MBD has not yet been made in either the coalescent nor the branching process literature.

When comparing the theoretical expected distributions discussed here with experimental data, two further factors come into play: noise and sampling. The impact of noise on bulk whole genome or exome sequencing data has been investigated at length [12,54], where increasing the depth of coverage in sequencing and filtering out the possible false mutations with single reads help to reduce noise. However, single-cell DNA sequencing faces much higher levels of noise due to the limited amount of DNA in single cells and consequently high amplification errors and bias generated in multiple polymerase chain reactions (PCR) [55–57]. While bioinformatic tools have been developed to handle the noise in calling mutations from single-cell DNA sequencing data [57], obtaining reliable single-cell MBDs directly from such data remains challenging. Consequently, constructing single-cell phylogenies and DDs can be also difficult when using single-cell data generated by PCR-based sequencing technologies. While a wide application of theoretical tools developed herein would rely on the improvement of technologies to generate more reliable data, there are a few designed experiments providing robust “single-cell” MBDs through whole-genome sequencing of single cell-derived colonies [4,58]. Sequencing errors are avoided by only using clonal mutations with high frequencies in single cell-derived colonies, which are private mutations in single ancestor cells back in the evolutionary time. Pulling together those mutations across all single cell-derived colonies from the same donor, reliable “single-cell” mutation burden distributions can be generated with the sampling size as the number of sequenced colonies.

Next, the effect of the sampling size on the SFS is well-documented: following the aforementioned coalescent approach of Lambert [50], Dinh *et al*. [48] derived hypergeometric terms in the SFS under sampling. In addition, Durrett (see Theorem 3 of [21]) approximated the impact of sampling on the SFS, an approach that Stein and Werner [59] have recently used to model cancer treatment and its impact on genetic heterogeneity within a tumour. The MBD, however, does not suffer from the same sampling distortions as the SFS: Moeller, Mon Père *et al*. [19] demonstrated that the MBD provides a way of inferring evolutionary parameters regardless of sample size. They showed that sampling increases the noise, resulting in higher errors, but that the expectations provided by the inferences remain unchanged [19].

Our analysis holds for a single clone. Neutral subclones can be identified in our dynamic matrices as follows: rows (corresponding to cells) with an entry of 1 in a particular column (corresponding to a given mutation *m*) form a subclone, all of whose cells possess mutation *m*. We can therefore extract, from the matrices, summary statistics for that subclone. To see that we can recover the expected analytical distribution, consider the following argument. Each division event in the branching process gives rise to two new, identical processes, with the daughter cells acting as progenitors, and their mutations already clonal within their sub-processes. Let *m* be the mutation that defines a subclone (that is, all members of the subclone possess mutation *m*); the number of sites at which this mutation occurs in the population is the population size $N_{\text{sub}}$ of the subclone. We then simply modify our expected distributions by conditioning on the population size being $N_{\text{sub}}$ rather than the total population size (up to possible sampling effects, as previously discussed). For example, by estimating the number of mutations *M*_0_ possessed by the progenitor cell of the subclone, we can shift the MBD correspondingly, increasing all mutational burdens by *M*_0_.

A natural extension of our work is to consider clonal competition, where different subclones have different fitnesses. In the cancer context, this might correspond to a subclone that is resistant to treatment; see [59] for details, for example. While the two-type branching process, i.e. a branching process containing a wild-type and a differently-fit mutant type, has been solved by Antal and Krapivsky [60,61], their methods do not allow for the accrual of many neutral mutations, as is the goal of our analysis. Here, we can include clonal competition by labelling cells with an index *n*, where wild-type cells are characterised by *n* = 0 and mutants have *n* = 1. We would thus allow transitions from *n* = 0 to *n* = 1 during division events and let birth and death probabilities be type-dependent, which makes the clones have different fitnesses. Our approach then produces coupled (via *n*) recurrence relations, whose solutions are not tractable with the current methods (see S1 Appendix for further discussion).

Intuitively, we would think that the explicit single-cell MBD results from both the DD and the extra stochasticity arising from the mutational distribution at each past cell division (the internal nodes of the cell lineage tree). Surprisingly, we found that the latter nodal stochasticity does not play a large role in the MBD. While there is certainly higher variance in the MBD than in the DD, as evidenced by Fig 4b and 4c, the shapes of the two distributions remain similar and we can construct the MBD based on the DD and μ, the mean value of number of mutations acquired per cell in each past cell division. The derivation from Eq (8) to Eq (9) demonstrates that only the mean of the mutational distribution matters when obtaining the MBD, rather than its higher moments. We further tested this conclusion by applying other mutational distributions than the Poisson in stochastic simulations, which lead to the same predicted MBD, as shown in Fig B of S1 Appendix. Employing the binning procedure described in Eq (A27) of S1 Appendix allows us to retrace our steps from the MBD to the DD, which reinforces that it is only the mean of the mutational distribution that is of critical importance to the shape of the MBD, not the exact form of the distribution. By considering the variances of the two distributions, we note that the variance in the single-cell MBD itself is growing while that of the mutational distribution is fixed. We thus expect that after sufficient events, the former will dominate.

We showed that the expected mutational burden for an arbitrary cell in a population (the mean of the MBD) increases logarithmically with the step count *i* in our model (see Propositions G and H of S1 Appendix). In Moeller, Mon Père *et al*.’s continuous-time framework, this mean is shown to be the product of the expected number of divisions in the cell’s past and the mutational mean μ [19], much as we have argued in Fig 4 for our conversion from the DD to the MBD. Under their intuitive assumption of mutation burdens arising from a compound Poisson distribution, the variance of the MBD is dependent on the means and the variances of the DD and the mutational (Poisson) distribution [19], whereas our derivations and simulations show that only the mean of the mutational distribution plays a significant role, not its higher moments.

Knowledge of the connection between the DD and the MBD also provides a means of evaluating the divisions in a cell’s history. By reversing the argument in Fig 4, MBD data can provide the distribution of divisional histories in a cell population, without resorting to direct measurements (for example, via telomere shortening [62]).

While single-cell sequencing is still in its adolescence, grappling with hurdles such as trade-offs between sequencing noise, sample size and cost [63,64], there is a growing need and theoretical gap for mathematical and computational machinery to handle the vast quantities of data being produced [23,46]. Our model serves as a new framework to integrate single-cell and bulk information, and shows how various distributions of accumulated mutations are linked through the same stochastic process.

## Methods

Besides the analysis described in the Results section, we employed a modified Gillespie algorithm to stochastically simulate our system and verify our expressions [65]. The original Gillespie formulation is used to simulate (in a statistically exact manner) continuous-time reactions that have specified rates within one or multiple populations. Rather than independently drawing an exponentially-distributed random number for each reaction (here the reactions would be birth and death within the single population of cells), the Gillespie algorithm leverages the fact that the *time until the first reaction occurs* is also exponentially-distributed, with rate equal to the sum of the rates of all of the reactions. The algorithm evolves by drawing one such number, then randomly selecting (proportional to their rates) *which* reaction takes place at that time, before updating the populations.

In our discrete-time model, we need only draw the second of these random numbers, determining whether a birth or death occurs at the given step. The cell that is dividing or dying is then (uniformly, since mutations are neutral) randomly selected, replicating itself or being removed from the system, respectively. If the event was a birth, new mutations are added to the two daughter cells according to the mutational distribution considered (we use a Poisson distribution unless otherwise mentioned, such as in S1 Appendix).

## Supporting information

S1 Appendix Further mathematical proofs and discussion. (PDF)
