# Supplementary material for: Single-cell mutational burden distributions in birth–death processes
Source: PLoS Comput Biol. 2025 Jul 7;21(7):e1013241. doi: 10.1371/journal.pcbi.1013241 (PMC12266427; doi:10.1371/journal.pcbi.1013241)
Supplement: S1 Appendix — (PDF) [file pcbi.1013241.s001.pdf]

S1 Appendix

We first derive results about the expected population size described by the Markov chain depicted in Fig 1a and its probability of extinction, which allow us to make rigorous our main text statement that all of our expectations are conditioned on survival. We then prove several results from the main text. Next, we make additional statements about the mutational occurrences C_i defined by Eq (1) and the number of unique mutations M_i using our recurrence relation approach. We then comment on what happens if other (than Poisson) mutational distributions are used, and we define a conversion from a single-cell mutational burden distribution (MBD) to a division distribution (DD). Finally, we briefly discuss the case of two clones. Table A summarises the new notation for S1 Appendix.

**Symbol**	**Description**
γ_z	Extinction probability for a birth–death process starting at $N_0 = z$
$u_{z,n}(p, q; a)$	Random walk absorption probability; see Proposition C for full definition
T	Markov chain transition matrix, with entries $T_{m,n} = \mathbb{P}[N_i = n \mid N_{i-1} = m]$
$\zeta_{k,i}$	coefficient equal to $1 - (i-k)/(k+1)$ for $\lfloor (i+1)/2 \rfloor \leq k \leq i$
C_i	Number of mutational occurrences at step i: the sum of the entries of Y_i

Table A. Notation used in S1 Appendix, in addition to those in Table 1.

Extinction probabilities and expected population sizes

Consider the Markov chain on a finite state space depicted by Fig 1a.

Proposition A. *The extinction probability for the birth–death process starting at $N_0 = z$ with $\beta \neq \frac{1}{2}$ is*

$$\mathbb{P}[N_i = 0 \text{ for some } i \geq 0 \mid N_0 = z] = \gamma_z = \frac{(\delta/\beta)^N - (\delta/\beta)^z}{(\delta/\beta)^N - 1}.$$

Proof. Note that the $\beta = \delta = \frac{1}{2}$ case (which is not relevant for our analysis because we assume $\beta > \delta$ can be treated separately without much difficulty. See pages 344–345 of Chapter XIV,2 of [1] for both proofs. □

Remark. By symmetry, we note that the probability to absorb into the N state (having started at $N_0 = z$) is equal to $1 - \gamma_z$, as can be seen by making the change of variables

$$\{\beta, \delta, z\} \rightarrow \{\delta, \beta, N - z\}, \tag{A1}$$

which allows us to view the Markov chain in reverse, in a sense. Therefore we conclude that only on a set of zero probability does this Markov chain not reach an absorbing state. Sometimes, we will consider the large-population limit $N \to \infty$, where then the extinction probability is simply $(\delta/\beta)^z$. For large enough i, we then obtain the approximation for the survival probability $\mathbb{P}[N_i > 0 \mid N_0 = 1] \simeq 1 - \delta/\beta$.

Remark. A second interpretation of this Markov chain is of a biased random walk on the integers $\{0, \ldots, N\}$, where moving left and right induces the binomial-like distribution seen in Eq (A2). This specific set-up with absorbing boundaries is known as the gambler's ruin problem; the change of variables in Eq (A1) is analogous to putting ourselves in the other gambler's shoes. Feller [1] and many other books on probability theory give many more details than we discuss here.

We can now describe the expected value of this Markov chain at step i. We assume that N is large enough; that is, that the maximum population size $i + 1$ (having started at $N_0 = 1$) is less than N.

Proposition B. *The expected population at step i of the birth–death process in Fig 1a is*

$$\mathbb{E}[N_i \mid N_0 = 1] = \sum_{\substack{n=1 \\ i-n \text{ odd}}}^{i+1} \frac{n^2}{i+1} \binom{i+1}{\frac{i-(n-1)}{2}} \beta^{\frac{i+(n-1)}{2}} \delta^{\frac{i-(n-1)}{2}}, \tag{A2}$$

and when conditioned on survival, we have $\mathbb{E}[N_i \mid N_0 = 1, N_i > 0] = \mathbb{E}[N_i \mid N_0 = 1] / \mathbb{P}[N_i > 0 \mid N_0 = 1]$.

Proof. Consider the transition matrix T (independent of $i \geq 1$ by the homogeneity of our Markov chain):

$$T = \begin{pmatrix} 1 & 0 & 0 & \cdots & 0 & 0 \\ \delta & 0 & \beta & \cdots & 0 & 0 \\ 0 & \delta & 0 & \cdots & 0 & 0 \\ \vdots & \vdots & \vdots & \ddots & \vdots & \vdots \\ 0 & 0 & 0 & \cdots & 0 & \beta \\ 0 & 0 & 0 & \cdots & 0 & 1 \end{pmatrix} \quad \text{for entries} \quad T_{m,n} = \mathbb{P}\left[N_i = n \,|\, N_{i-1} = m\right].$$

A property of stochastic Markov matrices is that the entries of the powers of the matrix represent the multi-step probabilities: $T^i_{m,n} = \mathbb{P}\left[N_i = n \,|\, N_0 = m\right]$ (this can be simply proven, or concluded from the Chapman-Kolmogorov equations). We are interested in the first row of these matrices T^i: that is, the case $m = 1$. We will consider $N > i + 1$, so that the maximum attainable population in i steps (having started at $N_0 = 1$) is still less than the maximal and absorbing state N.

Note that the survival probability $\mathbb{P}\left[N_i > 0 \,|\, N_0 = 1\right]$ is thus

$$\mathbb{P}\left[N_i > 0 \,|\, N_0 = 1\right] = \sum_{n=1}^{i+1} T^i_{1,n} = 1 - T^i_{1,0}. \tag{A3}$$

This sum as $i \to \infty$ is $1 - \delta/\beta$, as noted in the remark following Proposition A.

For $1 \leq i < N - 1$ the entries of the matrix powers satisfy the following recurrence relation

$$T^{i+1}_{1,n} = T_{n-1,n}T^i_{1,n-1} + T_{n+1,n}T^i_{1,n+1} = \beta T^i_{1,n-1} + \delta T^i_{1,n+1} \quad \text{for} \quad 1 < n < N - 1, \tag{A4}$$

along with $T^{i+1}_{1,0} = T^i_{1,0} + \delta T^i_{1,1}$, $T^{i+1}_{1,1} = \delta T^i_{1,2}$, $T^{i+1}_{1,N-1} = \beta T^i_{1,N-2}$ and $T^{i+1}_{1,N} = \beta T^i_{1,N-1} + T^i_{1,N}$.

For $n > 0$, consider the transition probability $T^i_{1,n}$: undergoing births or deaths, i steps are taken to progress from state 1 to state n. So, the sum of powers of β and δ should be i and their difference should be $n - 1$. The integer coefficients turn out to form the Catalan triangle (see the proof of Corollary B for an interpretation of this fact), so we obtain the ansatz

$$T^i_{1,n} = \frac{n}{i+1}\binom{i+1}{\frac{i-(n-1)}{2}}\beta^{\frac{i+(n-1)}{2}}\delta^{\frac{i-(n-1)}{2}} \quad \text{for} \quad 1 \leq n \leq i + 1 < N, \tag{A5}$$

where the binomial coefficients are defined to be zero if i and n have the same parity, since it is impossible to reach an odd (respectively even) destination n in an even (respectively odd) number of steps having started from 1. It is straightforward to show that the expression Eq (A5) satisfies the recurrence Eq (A4):

$$\begin{aligned}
\beta T^i_{1,n-1} + \delta T^i_{1,n+1} &= \beta\frac{n-1}{i+1}\binom{i+1}{\frac{i-(n-2)}{2}}\beta^{\frac{i+(n-2)}{2}}\delta^{\frac{i-(n-2)}{2}} + \delta\frac{n+1}{i+1}\binom{i+1}{\frac{i-n}{2}}\beta^{\frac{i+n}{2}}\delta^{\frac{i-n}{2}} \\
&= \frac{i!\beta^{\frac{i+n}{2}}\delta^{\frac{i-n}{2}+1}}{\left(\frac{i-n}{2}\right)!\left(\frac{i+n}{2}\right)!}\left(\frac{n-1}{\frac{i-n}{2}+1} + \frac{n+1}{\frac{i+n}{2}+1}\right) \\
&= \frac{n(i+1)!}{\left(\frac{i-n}{2}+1\right)!\left(\frac{i+n}{2}+1\right)!}\beta^{\frac{i+n}{2}}\delta^{\frac{i-n}{2}+1} \\
&= T^{i+1}_{1,n}.
\end{aligned}$$

Using Eq (A5), the expected population size non-conditioned on survival is then

$$\mathbb{E}\left[N_i \,|\, N_0 = 1\right] = \sum_{n=0}^{i+1} n\mathbb{P}\left[N_i = n \,|\, N_0 = 1\right] = \sum_{\substack{n=1 \\ i-n \text{ odd}}}^{i+1} \frac{n^2}{i+1}\binom{i+1}{\frac{i-(n-1)}{2}}\beta^{\frac{i+(n-1)}{2}}\delta^{\frac{i-(n-1)}{2}},$$

as desired. Conditioning on survival, we find

$$\begin{aligned}\mathbb{E}\left[N_i \,|\, N_0 = 1, N_i > 0\right] &= \sum_{n=0}^{i+1} n\mathbb{P}\left[N_i = n \,|\, N_0 = 1, N_i > 0\right] \\ &= \sum_{n=0}^{i+1} n\frac{\mathbb{P}\left[N_i = n \cap N_i > 0 \,|\, N_0 = 1\right]}{\mathbb{P}\left[N_i > 0 \,|\, N_0 = 1\right]} \\ &= \sum_{n=1}^{i+1} n\frac{\mathbb{P}\left[N_i = n \,|\, N_0 = 1\right]}{\mathbb{P}\left[N_i > 0 \,|\, N_0 = 1\right]} \\ &= \frac{\mathbb{E}\left[N_i \,|\, N_0 = 1\right]}{\mathbb{P}\left[N_i > 0 \,|\, N_0 = 1\right]}.\end{aligned}$$

□

Corollary A. *For k the number of births out of $i \leq 2k$ trials, we have*

$$T^i_{1,k-(i-k)+1} = \zeta_{k,i}\mathbb{P}\left[X = k\right], \tag{A6}$$

where $X \sim Binom(i, \beta)$ is a binomially-distributed random variable with probability β of success and $\zeta_{k,i}$ is defined for $\lfloor (i+1)/2 \rfloor \leq k \leq i$ by

$$\zeta_{k,i} = 1 - \frac{i-k}{k+1} \in (0, 1]. \tag{A7}$$

Proof. Write k for the number of births out of the i trials, so that $n = k - (i-k) + 1$. Then, Eq (A5) becomes

$$T^i_{1,n} = \frac{2n}{i+n+1}\mathbb{P}\left[X = \frac{i+(n-1)}{2}\right] = \left(1 - \frac{i-k}{k+1}\right)\mathbb{P}\left[X = k\right].$$

□

Remark. By itself, $\mathbb{P}[X = k]$ gives the probability of k successes out of i trials, where the successes can occur anywhere. The inclusion of a factor of $\zeta_{k,i}$ in Eq (A6) thus describes the case where the number of successes (births) must outweigh the number of failures (deaths) after any of the first $1 \leq i' \leq i$ trials had the experiment ended there, since we require the population to be non-extinct at each step. For example, we want the ordered sequence *bbdd* of births (b) and deaths (d) to contribute to $T^4_{1,1}$, but we do not want to count the sequence *bddb*, as the final b in the latter is not sensible, since the population is extinct after the third step. To formalise this, we state the following definitions and ensuing corollary.

A (p, q)-birth–death sequence is a word made from p letters b and q letters d, and a non-empty word obtained by removing (possibly zero) letters from the end of the original word is called a truncation. For example, *bbdbd* and *bbdb* are both truncations of the $(3, 2)$-birth–death sequence *bbdbd*. A (p, q)-birth–death sequence is called surviving if none of its $p + q$ truncations contain strictly more ds than bs. We then have the following interpretation of the coefficients $\zeta_{k,i}$.

Corollary B. *The proportion of surviving $(k, i-k)$-birth–death sequences is $\zeta_{k,i}$.*

Proof. By observation, the binomial coefficient $\binom{i}{k}$ is the total number of $(k, i-k)$-birth–death sequences. On the other hand, another interpretation of the Catalan triangle coefficients $\zeta_{k,i}\binom{i}{k}$ arising in Eq (A5) is the number of i-step walks from $(0, 1)$ to $(i, k-(i-k)+1)$, where each step goes from (x, y) to $(x+1, y \pm 1)$ and the walk stays in the positive quadrant. Therefore, they count the number of surviving $(k, i-k)$-birth–death sequences. □

Remark. Indeed, the definition Eq (A7) of $\zeta_{k,i}$ resembles the large-i survival probability $1 - \delta/\beta$, since $i - k$ is the number of deaths and $k + 1$ is one more than the number of births. Thus the expression Eq (A6) can be interpreted as this 'survival probability' $\zeta_{k,i}$ multiplied by a binomial distribution, which does not see the absorbing boundary at 0.

Now, we can use our new expression Eq (A6) for the transition probabilities to compare the exact expected population with an intuitive linear approximation.

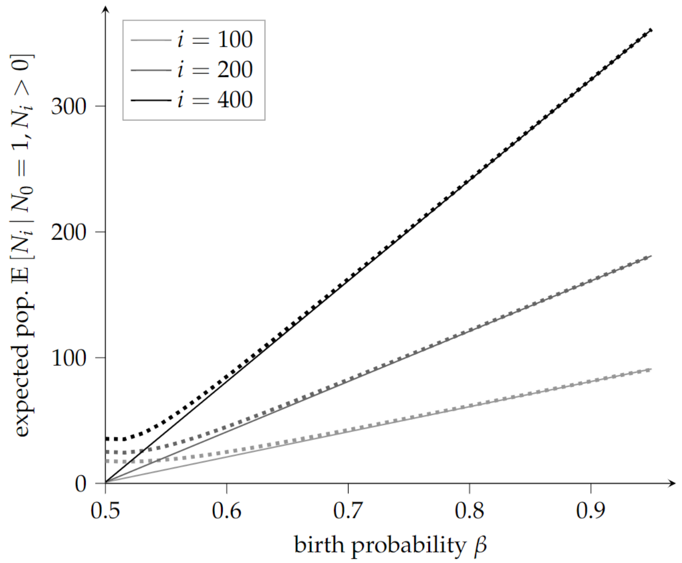

Fig A. Plot of the expected population size conditioned on survival $\mathbb{E}[N_i \mid N_0 = 1, N_i > 0]$ versus the birth probability β for different numbers of steps $i = 100$ (pale grey), $i = 200$ (dark grey) and $i = 400$ (black). The dotted lines show the exact expression from Proposition B, and the solid lines are the linear approximation $(\beta - \delta)i + 1$, showing excellent agreement for low death $\delta \ll \beta$ regardless of i.

Remark. The sums in the expected population Eq (A2) and the survival probability Eq (A3) only involve terms $1 \le n \le i + 1$ where $i - n$ is odd. When $n = k - (i - k) + 1$, this translates to summing over integers $\lfloor (i+1)/2 \rfloor \le k \le i$. Using Eq (A3) and Eq (A6), the expected population size conditional on survival is then

$$\mathbb{E}[N_i \mid N_0 = 1, N_i > 0] = \frac{\sum_{k=\lfloor \frac{i+1}{2} \rfloor}^{i} (k - (i-k) + 1)\zeta_{k,i} \mathbb{P}[X = k]}{\sum_{k'=\lfloor \frac{i+1}{2} \rfloor}^{i} \zeta_{k',i} \mathbb{P}[X = k']}. \tag{A8}$$

Remark. Consider a naïve derivation of the expected population size, conditioning on step $i - 1$

$$N_i = \beta(N_{i-1} + 1) + \delta(N_{i-1} - 1) = N_{i-1} + \beta - \delta = \cdots = (\beta - \delta)i + 1, \tag{A9}$$

and taking expectations of both sides, where we have abused notation by writing β and δ instead of $\mathbb{1}_{\{R=1\}}$ and $\mathbb{1}_{\{R=-1\}}$, respectively. This is an appropriate linear approximation in the limit of low death, but does not correctly condition on survival, as shown by the discrepancy for low β (see Fig A).

Remark. A heuristic derivation of the linear approximation Eq (A9) from the exact expression Eq (A8) is the following. For large i, the probability mass function of the binomial distribution tends to a function with mean $i\beta = k$, the expected number of births in i steps, and variance $i\beta(1 - \beta)$. When computing the sums in Eq (A8) for large i, consider the dominant $k = i\beta$ term (where we gloss over whether or not $i\beta$ is an integer) as the primary contribution to the sum:

$$\frac{\sum_k (k - (i-k) + 1)\zeta_{k,i} \mathbb{P}[X = k]}{\sum_{k'} \zeta_{k',i} \mathbb{P}[X = k']} \simeq \frac{(i\beta - (i - i\beta) + 1)\, \zeta_{i\beta,i}}{\zeta_{i\beta,i}} = (2\beta - 1)i + 1,$$

recovering Eq (A9). It is also worth noting that $\zeta_{i\beta,i} = 1 - (i - i\beta)/(i\beta + 1)$ coincides with the large-i survival probability $1 - \delta/\beta$, supporting our intuition that $\zeta_{k,i}$ can be thought of as a survival probability of sorts, as anticipated by Corollary B and the remark that follows it.

The exact survival probability at step i is given explicitly by Eq (A3) and Eq (A5). A related result from Feller [1] gives the distribution of times until the Markov process goes extinct.

Proposition C (Feller). *Consider a random walk on the integers* $\{0, \ldots, a\}$ *(with absorption on both end points) beginning at* z *with probability* $p \in (0, 1)$ *of moving right and* $q = 1 - p$ *of moving left. The probability of absorption on the* 0 *boundary at step* n *is*

$$u_{z,n}(p, q; a) = 2^n p^{(n-z)/2} q^{(n+z)/2} \frac{1}{a} \sum_{k=1}^{a-1} \cos^{n-1} \frac{\pi k}{a} \sin \frac{\pi k}{a} \sin \frac{\pi z k}{a}. \tag{A10}$$

Proof. See pages 349–353 of Chapter XIV,4 of [1]. □

Remark. Sending $a \to \infty$ in Eq (A10) provides a simplification [1]:

$$\lim_{a \to \infty} u_{z,n}(p, q; a) = \frac{z}{n} \binom{n}{\frac{n-z}{2}} p^{\frac{n-z}{2}} q^{\frac{n+z}{2}}, \tag{A11}$$

where the binomial coefficient implicitly assumes that n and z have the same parity. This is the case of only a single absorbing boundary at 0, as in our considered birth–death case. The similarity between Eq (A5) and Eq (A11) allows to state that $u_{1,i+1} = \delta T^i_{1,1}$, which is reasonable as the former is the probability of going extinct on step $i + 1$ and the latter is the probability of being at 1 on step i multiplied by the death probability δ. We can then use Eq (A11), the previous argument and Corollary A to find alternate expressions for the survival probability, where $X \sim \text{Binom}(i, \beta)$ as usual:

$$\mathbb{P}[N_i > 0 \,|\, N_0 = 1] = 1 - \sum_{\substack{n=1 \\ n \text{ odd}}}^{i} u_{1,n}(\beta, \delta; N) = 1 - \delta \sum_{\substack{m=0 \\ m \text{ even}}}^{i-1} T^m_{1,1} = 1 - \delta \sum_{\ell=0}^{\lfloor (i-1)/2 \rfloor} \frac{\mathbb{P}[X = \ell]}{\ell + 1}.$$

Proofs of recurrence relations results

Our first-order expected distributions depend on Taylor expansions of expected values of functions of random variables, along with the expected population size low-death approximation Eq (A9).

Lemma A. *Consider* A *and* B *independent random variables with finite moments and let* $f(A, B)$ *be a function that is* C^3 *near the point* $(\mathbb{E}[A], \mathbb{E}[B])$*. The second-order Taylor expansion of* $\mathbb{E}[f(A, B)]$ *about* $(\mathbb{E}[A], \mathbb{E}[B])$ *is*

$$\mathbb{E}[f(A, B)] = f(\mathbb{E}[A], \mathbb{E}[B]) + \frac{1}{2} f_{AA} \operatorname{var}(A) + f_{AB} \operatorname{cov}(A, B) + \frac{1}{2} f_{BB} \operatorname{var}(B) + O^3, \tag{A12}$$

where the second derivatives $f_{ij} = \partial_i \partial_j f$ *are evaluated at* $(\mathbb{E}[A], \mathbb{E}[B])$ *and* O^3 *is big-O notation for any cubic terms (which vanish as* A *and* B *approach their means).*

Proof. Eq (A12) is simply the second-order Taylor expansion about $(\mathbb{E}[A], \mathbb{E}[B])$ of

$$\mathbb{E}[f(A, B)] = \mathbb{E}[f(\mathbb{E}[A] + (A - \mathbb{E}[A]), \mathbb{E}[B] + (B - \mathbb{E}[B]))],$$

where the first-order terms vanish since the linearity of the expectation allows one to write them as proportional to $\mathbb{E}[A - \mathbb{E}[A]] = 0$ (and likewise for B). See pages 165–167 of Chapter 4 of [2] for details, for example. □

Remark. The first-order expansion of $\mathbb{E}[f(A, B)]$ about $(\mathbb{E}[A], \mathbb{E}[B])$ is $f(\mathbb{E}[A], \mathbb{E}[B])$.

Corollary C. *For* $B \neq 0$, $\mathbb{E}[B] \neq 0$ *and* $f(A, B) = A/B$*, to second order about* $(\mathbb{E}[A], \mathbb{E}[B])$*, we have*

$$\mathbb{E}\left[\frac{A}{B}\right] \simeq \frac{\mathbb{E}[A]}{\mathbb{E}[B]} - \frac{\operatorname{cov}(A, B)}{\mathbb{E}[B]^2} + \frac{\mathbb{E}[A] \operatorname{var}(B)}{\mathbb{E}[B]^3} = \frac{\mathbb{E}[A]}{\mathbb{E}[B]} - \frac{\mathbb{E}[AB]}{\mathbb{E}[B]^2} + \frac{\mathbb{E}[A] \mathbb{E}[B^2]}{\mathbb{E}[B]^3}.$$

We can now derive the birth–death site frequency spectrum, conditioned on survival. Note that other methods of derivation exist in the literature; two prominent examples can be found in Gunnarsson et al. [3], which contains approaches in both the stochastic-time and stochastic-population regime, as well as in Cheek and Antal [4], who provide a simple proof that leverages the solution of the two-type branching process by Antal and Krapivsky [5, 6].

Proposition D. *Starting from one mutation-free progenitor cell, the birth–death process with new mutations arising with expectation μ has first-order (near $(\mathbb{E}[S_{j,i}], \mathbb{E}[N_i])$; again, this will hold when $S_{j,i}$ and N_i are close to their expected values) expected site frequency spectrum*

$$\mathbb{E}[S_{j,i} \mid N_0 = 1, N_i > 0] \simeq \sum_{j'=0}^{\infty} \frac{2\mu(\delta/\beta)^{j'}}{(j+j')(j+j'+1)} \mathbb{E}[N_i \mid N_0 = 1, N_i > 0]. \tag{A13}$$

Proof. As in the main text, we use the law of total expectation to write a recurrence relation for the SFS at step $i+1$ conditional on the SFS at step i:

$$\begin{aligned}\mathbb{E}[S_{j,i+1}] &= \mathbb{E}[\mathbb{E}[S_{j,i+1} \mid \{S_{j',i}\}_{j' \leq i}]] \\ &= \mathbb{E}\left[S_{j,i} + \mathbb{1}_{\{R=1\}}\left(-\frac{jS_{j,i}}{N_i} + \frac{(j-1)S_{j-1,i}}{N_i} + (U_1+U_2)\delta_{1,j}\right) + \mathbb{1}_{\{R=-1\}}\left(-\frac{jS_{j,i}}{N_i} + \frac{(j+1)S_{j+1,i}}{N_i}\right)\right],\end{aligned}$$

for $\delta_{\cdot,\cdot}$ the Kronecker delta symbol, and where we have omitted the conditioning on survival and the initial condition $N_0 = 1$ for brevity. The first term in each set of parentheses in the second line arises because any j-abundant mutations in the dividing (respectively dying) cell, the number of which is $jS_{j,i}/N_i$, will become $(j \pm 1)$-abundant and thus no longer contribute to the j-site. The second terms depict the corresponding contribution from any $(j \mp 1)$-abundant mutations in the dividing (respectively dying) cell, which will become j-abundant. The Kronecker delta source term represents the new (thus 1-abundant) mutations arising in any division, arising as independently-drawn Poisson variables $U_1, U_2 \sim \text{Pois}(\mu)$.

Remark. For $N_0 = 1$, by observation, the expected population size and SFS at step $i = 1$ are $\mathbb{E}[N_1] = 2\beta$ and $\mathbb{E}[S_{1,1}] = 2\mu\beta$, with the β factors dropping if we condition on survival.

In the pure-birth case, the missing telescoping calculation (for $Q_j = \mathbb{E}[S_{j,i}]/(i+1)$) in the main text is

$$Q_j = \frac{j-1}{j+1}Q_{j-1} = \frac{j-1}{j+1}\frac{j-2}{j}Q_{j-2} = \cdots = \frac{2}{j(j+1)}Q_1 = \frac{2\mu}{j(j+1)},$$

which is indeed Eq (A13) when $\delta = 0$ (as only the $j = 0$ term in the infinite sum remains). A proof similar to this was presented in [7].

Now, for the birth–death case: if next we consider only the first-order approximation from Eq (A12)

$$\mathbb{E}\left[\frac{S_{j,i}}{N_i} \,\middle|\, N_0 = 1, N_i > 0\right] \simeq \frac{\mathbb{E}[S_{j,i} \mid N_0 = 1, N_i > 0]}{\mathbb{E}[N_i \mid N_0 = 1, N_i > 0]} = X_j,$$

where the final equality is the same ansatz as before (reasonable given our knowledge from [3] that $\mathbb{E}[S_{j,i} \mid N_0 = 1, N_i > 0] \propto \mathbb{E}[N_i \mid N_0 = 1, N_i > 0]$). Again, we have a second-order non-homogeneous linear recurrence relation for X_j:

$$X_j\mathbb{E}[N_{i+1} \mid N_0 = 1, N_i > 0] - X_j\mathbb{E}[N_i \mid N_0 = 1, N_i > 0] = -jX_j + \beta(j-1)X_{j-1} + \delta(j+1)X_{j+1} + 2\mu\beta\delta_{1,j}.$$

For low death, where our first order approximation holds, from Fig A and Eq (A9) we note that the expected gain in population size in one time step is $\beta - \delta$. Thus the previous expression becomes

$$\delta(j+2)X_{j+2} - (\beta - \delta + j + 1)X_{j+1} + \beta jX_j = 0 \quad \text{for} \quad j \geq 1, \tag{A14}$$

where we have absorbed the source term into the boundary conditions $X_1 = C$ and $X_2 = \beta(C - \mu)/\delta$.

Remark. One way to proceed is to use a shift operator S (such that $SX_j = X_{j+1}$) and factorise Eq (A14), solving first for one root of the ensuing quadratic equation in S and then the other.

Instead, we follow Deijfen et al. [8] in solving a second-order recurrence relation of the form

$$(\alpha_2(j+2) + \beta_2)X_{j+2} + (\alpha_1(j+1) + \beta_1)X_{j+1} + (\alpha_0 j + \beta_0)X_j = 0, \tag{A15}$$

which, in our case Eq (A14), we have $\{\alpha_i\}_{i=0}^2 = \{\delta, -1, \beta\}$ and $\{\beta_i\}_{i=0}^2 = \{0, \delta-\beta, 0\}$. To solve Eq (A15) with appropriate boundary conditions we must find an interval $[a, b]$ and a function $h(t)$ such that

$$\left[t^j h(t)\left(\alpha_2 t^2 + \alpha_1 t + \alpha_0\right)\right]_a^b = 0 \quad \text{and} \quad \frac{h'(t)}{h(t)} = \frac{\beta_2 t^2 + \beta_1 t + \beta_0}{t(\alpha_2 t^2 + \alpha_1 t + \alpha_0)},$$

where $h(t)$ is defined up to a multiplicative constant determined by the boundary conditions. The solution to the recurrence relation Eq (A15) is then

$$X_j \propto \int_a^b t^{j-1} h(t) \mathrm{d}t. \tag{A16}$$

Still following Deijfen et al. [8], we find $[a, b] = [0, 1]$ and

$$h(t) = \frac{1-t}{\beta/\delta - t},$$

which is valid for $\delta > 0$. From a table of integrals [9] we have

$$\int_0^1 x^{\lambda-1}(1-x)^{\mu-1}(1-\xi x)^{-\nu}\mathrm{d}x = B(\lambda,\mu)\, {}_2F_1(\nu,\lambda;\lambda+\mu;\xi) \tag{A17}$$

for $\Re(\lambda) > 0$, $\Re(\mu) > 0$ and $|\xi| < 1$, where we have defined the beta function $B(w, y)$ as

$$B(w,y) = \int_0^1 s^{w-1}(1-s)^{y-1}\mathrm{d}s,$$

and the hypergeometric function ${}_2F_1(k, l; m; z)$ as

$${}_2F_1(k,l;m;z) = \sum_{n=0}^{\infty} \frac{(k)_n (l)_n}{(m)_n} \frac{z^n}{n!},$$

for the rising Pochhammer symbol $(q)_n$

$$(q)_n = \begin{cases} 1 & \text{if } n = 0, \\ q(q+1)\cdots(q+n-1) & \text{if } n > 0. \end{cases}$$

Substituting $\{\lambda, \mu, \nu, \xi\} \to \{j, 2, 1, \delta/\beta\}$ into Eq (A17) and momentarily ignoring a constant factor of δ/β since $h(t)$ defined up to a constant, Eq (A16) becomes

$$\begin{aligned} X_j &\propto \int_0^1 t^{j-1}\frac{1-t}{1-(\delta/\beta)t}\mathrm{d}t \\ &= B(j,2)\, {}_2F_1(1,j;j+2;\delta/\beta) \\ &= \left(\int_0^1 t^{j-1}(1-t)\mathrm{d}t\right)\sum_{n=0}^{\infty}\frac{(1)_n (j)_n}{(j+2)_n}\frac{(\delta/\beta)^n}{n!} \\ &= \left(\frac{1}{j} - \frac{1}{j+1}\right)\sum_{n=0}^{\infty}\frac{j(j+1)}{(j+n)(j+n+1)}\left(\frac{\delta}{\beta}\right)^n, \end{aligned}$$

which is Eq (A13) after cancelling the factor $j(j+1)$ and multiplying by 2μ (so that $h(t)$ matches the boundary condition $X_1 = \mathbb{E}[S_{1,1}]/\mathbb{E}[N_1] = \mu$). □

Remark. The integral Eq (A16) is found via different methods in [3] for the continuous time (stochastic-population) case, though with bounds $[0, 1 - 1/N]$. Thus in the large population limit these coincide, which aligns with our intuition that in the large population limit the continuous and discrete time cases should converge, as discussed in [3].

We now derive the division distribution in the pure-birth and birth–death cases.

Remark. For the expected pure-birth DD, the main text is simply missing an observation that the solution Eq (6) solves the recurrence relation Eq (5):

$$\begin{bmatrix} i+1 \\ \ell \end{bmatrix} \frac{2^\ell}{(i+1)!} = \left(i \begin{bmatrix} i \\ \ell \end{bmatrix} + \begin{bmatrix} i \\ \ell-1 \end{bmatrix} \right) \frac{2^\ell}{(i+1)!} = \frac{i}{i+1} \begin{bmatrix} i \\ \ell \end{bmatrix} \frac{2^\ell}{i!} + \frac{2}{i+1} \begin{bmatrix} i \\ \ell-1 \end{bmatrix} \frac{2^{\ell-1}}{i!}.$$

Proposition E. *For the birth–death process described in Fig 1a, the expected division distribution is approximated to first order (near* $(\mathbb{E}[D_{\ell,i}], \mathbb{E}[N_i])$*; again, this will hold when* $D_{\ell,i}$ *and* N_i *are close to their expected values) by*

$$\mathbb{E}[D_{\ell,i} \mid N_0 = 1, N_i > 0] \simeq \frac{\left[{i \atop \ell} \right] 2^\ell (1-\delta/\beta)^{-\ell}}{\sum_{\ell'=1}^{i} \left[{i \atop \ell'} \right] 2^{\ell'} (1-\delta/\beta)^{-\ell'}} \mathbb{E}[N_i \mid N_0 = 1, N_i > 0]. \tag{A18}$$

Proof. We employ our same approach as for the pure-birth case:

$$\begin{aligned} \mathbb{E}[D_{\ell,i+1}] &= \mathbb{E}[\mathbb{E}[D_{\ell,i+1} \mid \{D_{\ell',i}\}_{\ell' \le i}, N_{i+1} > 0]] \\ &= \mathbb{E}\left[D_{\ell,i} + \mathbb{1}_{\{R=1\}} \left(-\frac{1}{N_i} D_{\ell,i} + \frac{2}{N_i} D_{\ell-1,i} \right) + \mathbb{1}_{\{R=-1\}} \left(-\frac{1}{N_i} D_{\ell,i} \right) \right] \\ &\simeq \left(1 - \frac{1}{\mathbb{E}[N_i]} \right) \mathbb{E}[D_{\ell,i}] + \frac{2\beta}{\mathbb{E}[N_i]} \mathbb{E}[D_{\ell-1,i}], \end{aligned}$$

up to first order, where in the second line the impact of a cell dying is simply to reduce its contribution to the division distribution by its expected amount. As before, we have omitted the conditioning on survival and the initial condition $N_0 = 1$ for brevity.

Next, consider the ansatz

$$\mathbb{E}[D_{\ell,i}]_{\text{az}} = \frac{\left[{i \atop \ell} \right] (2\beta)^\ell (\beta-\delta)^{i-\ell}}{\prod_{i'=1}^{i-1} \mathbb{E}[N_{i'}]}, \tag{A19}$$

which satisfies the previous recurrence relation by noting that, as argued in the remark following Proposition B (as well as by Eq (A9) and Fig A), $\mathbb{E}[N_i \mid N_0 = 1, N_i > 0] - 1 \simeq (\beta - \delta) i$ in the low-death limit:

$$\begin{aligned} \mathbb{E}[D_{\ell,i+1}]_{\text{az}} &= \frac{\left[{i+1 \atop \ell} \right] (2\beta)^\ell (\beta-\delta)^{i+1-\ell}}{\prod_{i'=1}^{i} \mathbb{E}[N_{i'}]} \\ &= \left(i \begin{bmatrix} i \\ \ell \end{bmatrix} + \begin{bmatrix} i \\ \ell-1 \end{bmatrix} \right) \frac{(2\beta)^\ell (\beta-\delta)^{i+1-\ell}}{\mathbb{E}[N_i] \prod_{i'=1}^{i-1} \mathbb{E}[N_{i'}]} \\ &= \frac{\mathbb{E}[N_i] - 1}{\mathbb{E}[N_i]} \begin{bmatrix} i \\ \ell \end{bmatrix} \frac{(2\beta)^\ell (\beta-\delta)^{i-\ell}}{\prod_{i'=1}^{i-1} \mathbb{E}[N_{i'}]} + \frac{2\beta}{\mathbb{E}[N_i]} \begin{bmatrix} i \\ \ell-1 \end{bmatrix} \frac{(2\beta)^{\ell-1} (\beta-\delta)^{i-(\ell-1)}}{\prod_{i'=1}^{i-1} \mathbb{E}[N_{i'}]}. \end{aligned}$$

It remains to show that the ansatz Eq (A19) can be rewritten as Eq (A18). In this pursuit, we employ the following lemma.

Lemma B. *For* $x \neq 0$*, the unsigned Stirling numbers defined by Eq (7) satisfy*

$$\prod_{k=1}^{i} \left(\frac{k}{x} + 1 \right) = x^{-i} \sum_{k=1}^{i} \begin{bmatrix} i \\ k \end{bmatrix} (1+x)^k. \tag{A20}$$

Proof of Lemma B. Note that both expressions coincide to $1 + 1/x$ when $i = 1$; suppose they do for $1 \le i \le I$. For

$i = I + 1$, the right-hand side of Eq (A20) is

$$x^{-(I+1)} \sum_{k=1}^{I+1} \begin{bmatrix} I+1 \\ k \end{bmatrix} (1+x)^k = x^{-(I+1)} \sum_{k=1}^{I+1} \left(I \begin{bmatrix} I \\ k \end{bmatrix} + \begin{bmatrix} I \\ k-1 \end{bmatrix} \right) (1+x)^k$$
$$= x^{-(I+1)} \left(\sum_{k=1}^{I} I \begin{bmatrix} I \\ k \end{bmatrix} (1+x)^k + \sum_{k=1}^{I} \begin{bmatrix} I \\ k \end{bmatrix} (1+x)^{k+1} \right)$$
$$= \frac{1}{x} (I+1+x) \, x^{-I} \sum_{k=1}^{I} \begin{bmatrix} I \\ k \end{bmatrix} (1+x)^k$$
$$= \left(\frac{I+1}{x} + 1 \right) \prod_{k=1}^{I} \left(\frac{k}{x} + 1 \right),$$

where we have used the definition Eq (7) and the boundary conditions of the unsigned Stirling numbers of the first kind, resulting the left-hand expression of Eq (A20), concluding the proof by induction. □

Applying Lemma B with $x = 1/(\beta - \delta)$, Eq (A19) transforms into

$$\frac{\left[{i \atop \ell}\right] (2\beta)^\ell (\beta-\delta)^{i-\ell}}{\prod_{i'=1}^{i-1} \mathbb{E}\left[N_{i'}\right]} = \frac{\left[{i \atop \ell}\right] (2\beta)^\ell (\beta-\delta)^{i-\ell}}{(\beta-\delta)^i \sum_{\ell'=1}^{i} \left[{i \atop \ell'}\right] (1+1/(\beta-\delta))^{\ell'}} \mathbb{E}\left[N_i\right],$$

which is the desired expression Eq (A18) after the identifications

$$\frac{2\beta}{\beta-\delta} = 1 + \frac{1}{\beta-\delta} = 2\left(1 - \frac{\delta}{\beta}\right)^{-1}. \qquad \square$$

Remark. When the distributions of $D_{\ell,i}$ and N_i are not sufficiently clustered around their means (that is, when δ is not small), however, the first-order approximation for the division distribution is not sound. As both diverge as $i \to \infty$, we require their variances to grow more slowly than their means do.

Proposition F. *Under the conditions of the low-death approximation $\mathbb{E}\left[N_i \mid N_0 = 1, N_i > 0\right] \simeq (\beta-\delta)i + 1$ (see Fig A and surrounding remarks for details), the variance of the population size N_i for the birth–death process with $N_0 = 1$ satisfies*

$$\operatorname{var}(N_i) \simeq \left(1 - (\beta-\delta)^2\right) i,$$

and thus tends to 0 as $\delta \to 0$ (since then $\beta \to 1$), as expected.

Proof. We omit the conditioning on survival and the initial condition $N_0 = 1$ for brevity. Using our usual approach, we have

$$\mathbb{E}\left[N_{i+1}^2\right] = \mathbb{E}\left[\mathbb{E}\left[N_{i+1}^2 \,\middle|\, N_i\right]\right] = \mathbb{E}\left[N_i^2\right] + 2(\beta-\delta)\mathbb{E}\left[N_i\right] + 1 \simeq \mathbb{E}\left[N_i^2\right] + 2(\beta-\delta)\left((\beta-\delta)i + 1\right) + 1.$$

Solving the ensuing recurrence, we find

$$\mathbb{E}\left[N_i^2\right] \simeq (\beta-\delta)^2 i^2 + \left(-(\beta-\delta)^2 + 2(\beta-\delta) + 1\right) i + 1, \qquad \text{(A21)}$$

from which we subtract $\mathbb{E}\left[N_i\right]^2 \simeq ((\beta-\delta)i+1)^2$:

$$\operatorname{var}(N_i) \simeq (\beta-\delta)^2 i^2 + \left(-(\beta-\delta)^2 + 2(\beta-\delta) + 1\right) i + 1 - ((\beta-\delta)i+1)^2 = \left(1 - (\beta-\delta)^2\right) i. \qquad \square$$

Remark. There is an equivalent recurrence relation for the division distribution when expanded to second order using Corollary C, though it cannot be solved by elementary methods:

$$\begin{aligned}
\mathbb{E}\left[D_{\ell,i+1}\right] &= \mathbb{E}\left[D_{\ell,i}\right] - \mathbb{E}\left[\frac{D_{\ell,i}}{N_i}\right] + 2\beta\mathbb{E}\left[\frac{D_{\ell-1,i}}{N_i}\right] \\
&\simeq \mathbb{E}\left[D_{\ell,i}\right] - \left(\frac{\mathbb{E}\left[D_{\ell,i}\right]}{\mathbb{E}\left[N_i\right]} - \frac{\mathbb{E}\left[D_{\ell,i}N_i\right]}{\mathbb{E}\left[N_i\right]^2} + \frac{\mathbb{E}\left[D_{\ell,i}\right]\mathbb{E}\left[N_i^2\right]}{\mathbb{E}\left[N_i\right]^3}\right) \\
&\quad + 2\beta\left(\frac{\mathbb{E}\left[D_{\ell-1,i}\right]}{\mathbb{E}\left[N_i\right]} - \frac{\mathbb{E}\left[D_{\ell-1,i}N_i\right]}{\mathbb{E}\left[N_i\right]^2} + \frac{\mathbb{E}\left[D_{\ell-1,i}\right]\mathbb{E}\left[N_i^2\right]}{\mathbb{E}\left[N_i\right]^3}\right) \\
&= \mathbb{E}\left[D_{\ell,i}\right] - \frac{\mathbb{E}\left[D_{\ell,i}\right]}{\mathbb{E}\left[N_i\right]} + 2\beta\frac{\mathbb{E}\left[D_{\ell-1,i}\right]}{\mathbb{E}\left[N_i\right]} + \frac{\mathbb{E}\left[N_i^2\right]}{\mathbb{E}\left[N_i\right]^3}\left(2\beta\mathbb{E}\left[D_{\ell-1,i}\right] - \mathbb{E}\left[D_{\ell,i}\right]\right) \\
&\quad + \frac{1}{\mathbb{E}\left[N_i\right]^2}\sum_{\ell'=1}^{i}\left(\mathbb{E}\left[D_{\ell,i}D_{\ell',i}\right] - 2\beta\mathbb{E}\left[D_{\ell-1,i}D_{\ell',i}\right]\right),
\end{aligned}$$

where we used the linearity of expectation to simplify and have omitted all conditioning on survival and the initial condition $N_0 = 1$ for brevity, as usual. The first three terms in the final expression are those from the first-order expansion of Proposition E, and the next term can be simplified using Eq (A21). Despite this, the equation above for $\mathbb{E}\left[D_{\ell,i+1}\right]$ not only remains complicated but includes terms of the form $\mathbb{E}\left[D_{\ell,i}D_{\ell',i}\right]$, which prevent it from being solved using our usual recursive methods.

Mutational occurrences

Recall the definition of the number of mutational occurrences from Eq (1); we will write C_i for this quantity. Note that this is also equal to (up to renormalisation by the number of unique mutations M_i or the population N_i) the mean of the site frequency spectrum $\{S_{j,i}\}_j$ or the single-cell mutational burden distribution $\{B_{k,i}\}_k$, respectively. In the pure-birth case, we have the following result.

Proposition G. *In a pure-birth process with mutation rate μ starting from a single mutation-free progenitor cell, the expected number of unique mutations and mutational occurrences at step i are given by*

$$\mathbb{E}\left[M_i\right] = 2\mu i \quad \text{and} \quad \mathbb{E}\left[C_i\right] = 2\mu(i+1)\left(H_{i+1} - 1\right),$$

for $H_n = \sum_{k=1}^{n} k^{-1}$ the nth harmonic number. Note that $H_n = \gamma + \log n + \frac{1}{2n} + O(n^{-2})$ for $\gamma \approx 0.5772$ the Euler–Mascheroni constant, so the number of mutational occurrences C_i grows like $2\mu i \log i$ for large i.

Proof. With each birth, an expected 2μ new mutations are added to the population, so the result for $\mathbb{E}\left[M_i\right]$ is clear by observation. Taking the sum over j of the expected pure-birth SFS Eq (3) as in the definition $M_i = \sum_j S_{j,i}$, and using the linearity of expectation and $j^{-1}(j+1)^{-1} = j^{-1} - (j+1)^{-1}$ to telescope terms, we also obtain the desired expression $\mathbb{E}\left[M_i\right] = 2\mu i$.

Using the usual law of total expectation approach, the recurrence relation for C_i is

$$\mathbb{E}\left[C_{i+1}\right] = \mathbb{E}\left[\left(1 + \frac{1}{N_i}\right)C_i + 2\mu\right], \tag{A22}$$

since not only are 2μ new mutations arising with each division event, but all of the mutations in the dividing cell are duplicated. The proportion of cells with k mutations is $B_{k,i}/N_i$, so the expected number of mutations in the dividing cell is found by multiplying this proportion by k and summing over $1 \le k \le M_i$. This sum is exactly C_i/N_i by Eq (1), giving rise to the parenthetical term in Eq (A22).

Recalling that in the pure-birth case we have $N_i = i + 1$, Eq (A22) can be solved recursively in a similar manner to the pure-birth case of Proposition D: we divide both sides of Eq (A22) by $i + 2$, make a change of variables to $G_i = C_i/(i+1)$ such that $G_1 = C_1/2 = \mu$, telescope the G_i terms to find

$$G_{i+1} = G_1 + 2\mu\sum_{k=1}^{i}\frac{1}{k+2} = \mu + 2\mu\left(H_{i+2} - 1 - \frac{1}{2}\right) = 2\mu\left(H_{i+2} - 1\right),$$

which, after multiplying by $i+2$ and relabelling indices, is the desired expression. □

Proposition H. *For a birth–death process with mutation rate μ, the expected number of unique mutations $\mathbb{E}[M_i]$ to first order (near $(\mathbb{E}[S_{j,i}], \mathbb{E}[N_i])$) has the following form:*

$$\mathbb{E}[M_i] \simeq 2\mu \left(\beta - \frac{\beta(\beta-\delta)}{\delta} \log\left(1 - \frac{\delta}{\beta}\right)(i-1) \right) \tag{A23}$$

and the expected total mutational occurrences $\mathbb{E}[C_i]$ to first order (near $(\mathbb{E}[S_{j,i}], \mathbb{E}[N_i])$) obeys

$$\mathbb{E}[C_i] \simeq 2\mu\beta\mathbb{E}[N_i] \sum_{i'=1}^{i} \frac{1}{\mathbb{E}[N_{i'}]}, \tag{A24}$$

where all expectations are conditioned on survival and the initial condition $N_0 = 1$.

Remark. The expression Eq (A24) has no nice simplification even when the linear approximation of $\mathbb{E}[N_i]$ is used.

Proof. First we will find the expression Eq (A23) for $\mathbb{E}[M_i]$. Note that the previous contributions during birth events of the pure-birth case are simply weighted by β; the death events mean that 1-abundant mutations found in the dying cell (whose expected number is $\mathbb{E}[S_{1,i}/N_i]$, which we will expand to first order as before) vanish from the count at step $i+1$. Thus, the recurrence relation for $\mathbb{E}[M_i]$ is

$$\mathbb{E}[M_{i+1}] = \mathbb{E}\left[M_i + \mathbb{1}_{\{R=1\}} 2\mu - \mathbb{1}_{\{R=-1\}} \frac{S_{1,i}}{N_i} \right],$$

which is solved by summing over i and telescoping to find

$$\mathbb{E}[M_i] \simeq 2\mu\beta i - \delta \sum_{i'=1}^{i-1} \frac{\mathbb{E}[S_{1,i'}]}{\mathbb{E}[N_{i'}]} = 2\mu\beta i - \delta(i-1) \sum_{n=0}^{\infty} \frac{2\mu(\delta/\beta)^n}{(n+1)(n+2)}, \tag{A25}$$

where we have taken advantage of the fact that (under our approximations) $\mathbb{E}[S_{1,i}]/\mathbb{E}[N_i]$ is independent of i and substituted Eq (A13). A crude approximation would be this expression for $i'=1$: $\mathbb{E}[S_{1,1}]/\mathbb{E}[N_1] = \mu$. However, our approximations hold better for large i, so instead we compute

$$\sum_{n=0}^{\infty} \frac{w^n}{(n+1)(n+2)} = 1 - (1-w) \sum_{m=0}^{\infty} \frac{w^m}{m+2} = 1 + \frac{1-w}{w^2} - \frac{1-w}{w^2} \sum_{m=1}^{\infty} \frac{w^m}{m} = \frac{1}{w} + \frac{1-w}{w^2} \log(1-w),$$

which we substitute into Eq (A25) with $w = \delta/\beta$ to obtain

$$\mathbb{E}[M_i] \simeq 2\mu\beta i - 2\mu\delta(i-1) \left(\frac{\beta}{\delta} + \left(1 - \frac{\delta}{\beta}\right) \frac{\beta^2}{\delta^2} \log\left(1 - \frac{\delta}{\beta}\right) \right),$$

which can be rearranged to find Eq (A23).

To find the expression Eq (A24) for the total mutational occurrences, note that as before the birth contributions will be multiplied by $\mathbb{1}_{\{R=1\}}$; the death contributions to C_{i+1} include the previous quantity C_i, minus the number of mutations found in the dying cell, whose expected number is $\mathbb{E}[C_i/N_i]$, as argued in the proof of Proposition G. Thus, the recurrence relation satisfied by $\mathbb{E}[C_i]$ is

$$\mathbb{E}[C_{i+1}] = \mathbb{E}\left[\left(1 + \frac{\mathbb{1}_{\{R=1\}} - \mathbb{1}_{\{R=-1\}}}{N_i}\right) C_i + \mathbb{1}_{\{R=1\}} 2\mu \right].$$

We notice that by Eq (A9) the parenthetical term expands to first order to $\mathbb{E}[N_{i+1}]/\mathbb{E}[N_i]$, so by dividing by $\mathbb{E}[N_{i+1}]$ and solving for $\mathbb{E}[C_i]/\mathbb{E}[N_i]$ (by the same telescoping trick as in the proof of Proposition G), we obtain

$$\frac{\mathbb{E}[C_{i+1}]}{\mathbb{E}[N_{i+1}]} \simeq \frac{\mathbb{E}[C_1]}{\mathbb{E}[N_1]} + 2\mu\beta \sum_{i'=1}^{i} \frac{1}{\mathbb{E}[N_{i'+1}]} = \mu \left(1 + 2\beta \left(\sum_{i'=1}^{i+1} \frac{1}{\mathbb{E}[N_{i'}]} - \frac{1}{2\beta} \right) \right) = 2\mu\beta \sum_{i'=1}^{i+1} \frac{1}{\mathbb{E}[N_{i'}]},$$

which gives the desired expression after multiplying both sides by $\mathbb{E}[N_{i+1}]$ and relabelling indices. □

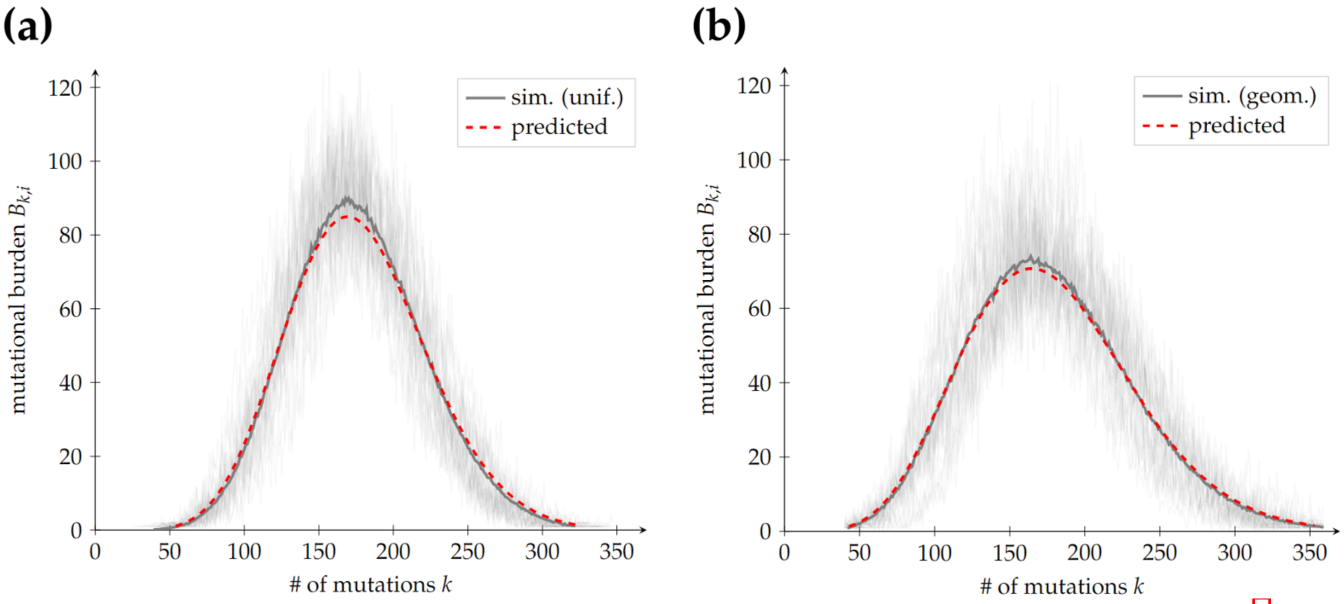

Fig B. Conversions from the division distribution to the single-cell mutational burden distribution as in Fig 4 of the main text, using other (than Poisson) mutational distributions, with means μ: **(a)** uniform distribution over the set $\{1, \ldots, 2\mu - 1\}$ and **(b)** geometric distribution with mean μ. Average (solid dark grey line) of 200 simulation realisations (representatives in solid pale grey lines) and the predicted MBD distribution converted from the DD (dashed red line).

Other mutational distributions

As noted in the main text, when other mutational distributions from the Poisson distribution are used in simulations, the conversion from the division distribution (DD) to the single-cell mutational burden distribution (MBD) described by Fig 4 still holds under some conditions. In Fig B, the mutational distributions used are Unif$\{1, \ldots, 2\mu - 1\}$ and Geom$(1/\mu)$, both of which have mean μ and are recaptured by the predicted distribution.

If, however, the number of new mutations U_1 and U_2 (where the indices refer to daughter cells) are drawn independently from a distribution with small support, such as the uniform distribution Unif$\{\mu - 1, \mu + 1\}$, then it is unsurprising that the smoothing procedure from a DD to a MBD of Fig 4 does not hold, since fewer possible mutational burdens are obtainable for cells. In the extreme case of the delta distribution Delta(μ), we simply obtain a scaling of the DD, where all cells have mutational burden equal to the product of the mutational mean and their division burden.

On the other hand, given an expected MBD, it is possible to recover the expected DD via a binning procedure. By histogramming the MBD with bins of width μ centred at integer multiples of μ (which results in the distribution supposing that exactly $U_1 = U_2 = \mu$ mutations were acquired with each division), we can then rescale by μ to obtain the DD. Symbolically,

$$\mathbb{E}\left[D_{\ell,i}\right] = \sum_{k \in \left[\left(\ell - \frac{1}{2}\right)\mu, \left(\ell + \frac{1}{2}\right)\mu\right)} \mathbb{E}\left[B_{k,i}\right].$$

In Fig 4a, this involves summing μ adjacent bins of the MBD, and then rescaling the x-axis by μ to obtain the DD. This is exactly the aforementioned distribution, obtained by using Delta(μ) as the mutational distribution.

Two clones with different fitnesses

Suppose there are two cell types, labelled by an index n, where $n = 0$ is the wild-type with unit fitness and $n = 1$ is the mutant with fitness $1 + s$ (where may be positive or negative). Rather than only having a single type dividing, accumulating mutations and dying, we now allow for one further reaction in our stochastic system: the asymmetric division of a wild-type into one wild-type and one mutant, occurring during the division of the wild-type cell with a

small probability $p \ll 1$; thus with probability $1-p$ the division of a wild-type cell results in two wild-type daughters. (We suppose that the symmetric division of one wild-type into two mutants is negligible.)

Antal and Krapivsky [5, 6] solved one such continuous-time two-type branching process, where the time is fixed and the population left stochastic; Kessler and Levine [10, 11] approached a similar problem in our fixed-population case, finding approximate expressions for the distribution of population between the types. Neither accounts for the accumulation of many neutral mutations, as we do here. Unfortunately, in the two-type scenario, our approach faces difficulties: namely, we obtain coupled recurrence relations that are not possible to solve with our methods.

Consider the simple pure-birth scenario, with two types as described above. Here, the birth probabilities $\{\beta^{(n)}\}_{n=0,1}$ of the two types are dependent on the population sizes $\{N_i^{(n)}\}_{n=0,1}$ (where now the total population $N_i = N_i^{(0)} + N_i^{(1)} = i+1$ is composed of the wild-type cells, $n=0$, and the mutant cells, $n=1$) of the types, or their proportions, as well as the step i:

$$\beta^{(0)} = \frac{N_i^{(0)}}{N_i^{(0)} + (1+s)\left(i+1-N_i^{(0)}\right)} \quad \text{and} \quad \beta^{(1)} = \frac{(1+s)\left(i+1-N_i^{(0)}\right)}{N_i^{(0)} + (1+s)\left(i+1-N_i^{(0)}\right)},$$

which sum to unity, as desired. Our random variable R now has three outcomes: symmetric birth of a wild-type cell into two wild-type daughters ($R=1$, occurring with probability $(1-p)\beta^{(0)}$); asymmetric birth of a wild-type cell into one wild-type daughter and one mutant daughter ($R=0$, occurring with probability $p\beta^{(0)}$); and symmetric birth of a mutant cell into two mutant daughters ($R=-1$, occurring with probability $\beta^{(1)} = 1-\beta^{(0)}$).

We define analogues to the SFS, DD and MBD, indexed by the type n; for example, $D_{\ell,i}^{(1)}$ is the number of mutant ($n=1$) cells having undergone ℓ divisions at step i. Conditioning on the three possible outcomes of the random variable R, the law of total expectation provides:

$$\begin{aligned}\mathbb{E}\left[D_{\ell,i+1}^{(0)}\right] &= \mathbb{E}\left[\mathbb{E}\left[D_{\ell,i+1}^{(0)} \,\middle|\, \left\{D_{\ell',i}^{(n)}\right\}_{n;\,\ell'\le i}\right]\right] \\ &= \mathbb{E}\left[\mathbb{1}_{\{R=1\}}\left(D_{\ell,i}^{(0)} - \frac{D_{\ell,i}^{(0)}}{i+1} + \frac{2D_{\ell-1,i}^{(0)}}{i+1}\right) + \mathbb{1}_{\{R=0\}}\left(D_{\ell,i}^{(0)} - \frac{D_{\ell,i}^{(0)}}{i+1} + \frac{D_{\ell-1,i}^{(0)}}{i+1}\right) + \mathbb{1}_{\{R=-1\}} D_{\ell,i}^{(0)}\right],\end{aligned}$$

for the wild-type cells, and

$$\mathbb{E}\left[D_{\ell,i+1}^{(1)}\right] = \mathbb{E}\left[\mathbb{1}_{\{R=1\}} D_{\ell,i}^{(1)} + \mathbb{1}_{\{R=0\}}\left(D_{\ell,i}^{(1)} + \frac{D_{\ell-1,i}^{(0)}}{i+1}\right) + \mathbb{1}_{\{R=-1\}}\left(D_{\ell,i}^{(1)} - \frac{D_{\ell,i}^{(1)}}{i+1} + \frac{2D_{\ell-1,i}^{(1)}}{i+1}\right)\right],$$

for the mutant cells. From these expressions it is clear that our previous methods will fail: first, they are coupled, due to the appearance of $D_{\ell-1,i}^{(0)}$ in the recurrence relation for the mutant cells; second, taking expectations of the form $\mathbb{E}\left[\mathbb{1}_{\{R=\bullet\}} D_{\ell,i}^{(n)}\right]$ requires delicacy, since the birth probabilities are themselves random variables; third, the birth probabilities change with each step i. Even if we use our first-order approximation $\mathbb{E}\left[f(A,B)\right] \simeq f(\mathbb{E}\left[A\right], \mathbb{E}\left[B\right])$ for both the products and the quotients of random variables, our recurrence relations take the form

$$\mathbb{E}\left[D_{\ell,i+1}^{(0)}\right] \simeq \left(1 - \frac{\mathbb{E}\left[\beta^{(0)}\right]}{i+1}\right)\mathbb{E}\left[D_{\ell,i}^{(0)}\right] + \frac{(2-p)\mathbb{E}\left[\beta^{(0)}\right]}{i+1}\mathbb{E}\left[D_{\ell-1,i}^{(0)}\right],$$

and

$$\mathbb{E}\left[D_{\ell,i+1}^{(1)}\right] \simeq \left(1 - \frac{1-\mathbb{E}\left[\beta^{(0)}\right]}{i+1}\right)\mathbb{E}\left[D_{\ell,i}^{(1)}\right] + \frac{2-(2-p)\mathbb{E}\left[\beta^{(0)}\right]}{i+1}\mathbb{E}\left[D_{\ell-1,i}^{(1)}\right],$$

which remain impenetrable. The expectation $\mathbb{E}\left[\beta^{(0)}\right]$ involves determining $\mathbb{E}\left[N_i^{(0)}\right]$, the expected number of wild-type cells at step i; this is what Antal and Krapivsky [5, 6] derived in the continuous-time, stochastic-population case. In conclusion, even in the relatively simple pure-birth case, deriving the DD for two clones of different fitnesses is a challenge beyond the scope of this paper. Similar difficulties are faced for our other summary statistics.

References

[1] W. Feller. *An Introduction to Probability Theory and Its Applications*. Vol. 1. John Wiley & Sons, 1968. Chap. XIV.

[2] H. Benaroya, S. M. Han, and M. Nagurka. *Probability Models in Engineering and Science*. Vol. 192. CRC press, 2005. Chap. 4.

[3] E. B. Gunnarsson, K. Leder, and J. Foo. "Exact site frequency spectra of neutrally evolving tumors: A transition between power laws reveals a signature of cell viability". In: *Theoretical Population Biology* 142 (2021), pp. 67–90.

[4] D. Cheek and T. Antal. "Mutation frequencies in a birth–death branching process". In: *The Annals of Applied Probability* 28.6 (2018), pp. 3922–3947.

[5] T. Antal and P. Krapivsky. "Exact solution of a two-type branching process: Clone size distribution in cell division kinetics". In: *Journal of Statistical Mechanics: Theory and Experiment* 2010.07 (2010), P07028.

[6] T. Antal and P. Krapivsky. "Exact solution of a two-type branching process: Models of tumor progression". In: *Journal of Statistical Mechanics: Theory and Experiment* 2011.08 (2011), P08018.

[7] M. E. Moeller et al. "Measures of genetic diversification in somatic tissues at bulk and single-cell resolution". In: *eLife* 12 (Jan. 2024), RP89780.

[8] M. Deijfen and M. Lindholm. "Growing networks with preferential deletion and addition of edges". In: *Physica A: Statistical Mechanics and Its Applications* 388.19 (2009), pp. 4297–4303.

[9] I. S. Gradshteyn and I. M. Ryzhik. *Table of Integrals, Series, and Products*. 7th ed. Academic Press, 2007.

[10] D. A. Kessler and H. Levine. "Large population solution of the stochastic Luria-Delbrück evolution model". In: *Proceedings of the National Academy of Sciences* 110.29 (2013), pp. 11682–11687.

[11] D. A. Kessler and H. Levine. "Scaling solution in the large population limit of the general asymmetric stochastic Luria-Delbrück evolution process". In: *Journal of Statistical Physics* 158.4 (2015), pp. 783–805.
